# An essential role for dNTP homeostasis following CDK-induced replication stress

**DOI:** 10.1242/jcs.226969

**Published:** 2019-03-25

**Authors:** Chen-Chun Pai, Kuo-Feng Hsu, Samuel C. Durley, Andrea Keszthelyi, Stephen E. Kearsey, Charalampos Rallis, Lisa K. Folkes, Rachel Deegan, Sarah E. Wilkins, Sophia X. Pfister, Nagore De León, Christopher J. Schofield, Jürg Bähler, Antony M. Carr, Timothy C. Humphrey

**Affiliations:** 1 CRUK-MRC Oxford Institute for Radiation Oncology, Department of Oncology, University of Oxford, ORCRB, Roosevelt Drive, Oxford, OX3 7DQ, UK; 2 Chemistry Research Laboratory, Department of Chemistry, University of Oxford, Oxford, OX1 3TA, UK; 3 Department of Surgery, Tri-Service General Hospital, National Defense Medical Centre, Taipei 114, Taiwan; 4 Genome Damage and Stability Centre, School of Life Sciences, University of Sussex, Falmer, Brighton, Sussex, BN1 9RQ, UK; 5 Department of Zoology, University of Oxford, Zoology Research & Administration Building, Mansfield Road, Oxford, OX1 3PS, UK; 6 Research Department of Genetics, Evolution & Environment, University College London, London, WC1E 6BT, UK; 7 School of Health, Sport and Bioscience, University of East London, Stratford Campus, E15 4LZ, London, UK

**Keywords:** Wee1, Histone H3K36 modification, *Schizosaccharomyces pombe*, Synthetic lethality, MBF, Set2, CDK

## Abstract

Replication stress is a common feature of cancer cells, and thus a potentially important therapeutic target. Here, we show that cyclin-dependent kinase (CDK)-induced replication stress, resulting from Wee1 inactivation, is synthetic lethal with mutations disrupting dNTP homeostasis in fission yeast. Wee1 inactivation leads to increased dNTP demand and replication stress through CDK-induced firing of dormant replication origins. Subsequent dNTP depletion leads to inefficient DNA replication, DNA damage and to genome instability. Cells respond to this replication stress by increasing dNTP supply through histone methyltransferase Set2-dependent MBF-induced expression of Cdc22, the catalytic subunit of ribonucleotide reductase (RNR). Disrupting dNTP synthesis following Wee1 inactivation, through abrogating Set2-dependent H3K36 tri-methylation or DNA integrity checkpoint inactivation results in critically low dNTP levels, replication collapse and cell death, which can be rescued by increasing dNTP levels. These findings support a ‘dNTP supply and demand’ model in which maintaining dNTP homeostasis is essential to prevent replication catastrophe in response to CDK-induced replication stress.

## Abstract (precis)

**Highlighted Article:** Replication stress is a source of genome instability. In this study in fission yeast, we describe a new role for the mitotic inhibitor Wee1 in suppressing replication stress and genome instability.

## INTRODUCTION

Replication stress, in which DNA replication forks stall, is a source of genome instability and a common feature of cancer cells (Gaillard et al., 2015). The ability to target such a hallmark of cancer cells is of significant therapeutic interest. Replication stress can result from multiple events including physical blockage of replication fork progression, deregulation of the replication initiation or elongation complexes or through deoxyribonucleoside triphosphate (dNTP) depletion (Dobbelstein and Sorensen, 2015; Zeman and Cimprich, 2014). Cells respond to such events by triggering checkpoint-dependent responses to facilitate DNA replication restart (Mazouzi et al., 2014). In humans, ATR and CHK1 (encoded by the *CHEK1* gene) are the primary kinases responsible for replication checkpoint activity, while in fission yeast (*Schizosaccharomyces pombe*) the Rad3 and Cds1 kinases play a predominant role, with Cds1 being redundant with Chk1 in this response (Boddy et al., 1998; Feijoo et al., 2001; Flynn and Zou, 2011; Lindsay et al., 1998). Unresponsive stalled forks can be subject to endonucleolytic cleavage by Mus81–Eme1, generating a DNA end, which is targeted for homologous recombination (HR) (Hanada et al., 2007; Roseaulin et al., 2008).

In fission yeast, dNTP synthesis is induced in response to replication stress and DNA damage by at least two distinct mechanisms (Guarino et al., 2014). Checkpoint activation promotes ubiquitin ligase complex Ddb1–Cul4^Cdt2^-dependent degradation of Spd1 (Cdt2 is the substrate adapter), an inhibitor of ribonucleotide reductase (RNR) (Håkansson et al., 2006; Liu et al., 2003), thereby promoting dNTP synthesis (Holmberg et al., 2005; Liu et al., 2005, 2003). In addition, checkpoint-dependent activation of the MluI cell cycle box (MCB)-binding factor (MBF) complex promotes transcription of genes encoding one or more MCB domains within their promoter regions, including *cdc22^+^*, the catalytic subunit of RNR, thereby promoting dNTP synthesis (Dutta et al., 2008).

The chromatin state plays an important role in modulating transcriptional responses. Set2 is a histone methyltransferase required for histone H3 lysine 36 (H3K36) mono-, di- and tri-methylation in yeast (Morris et al., 2005). Various functions have been ascribed to H3K36 methylation, including DNA repair (Pai et al., 2014) and checkpoint signalling (Jha and Strahl, 2014). Furthermore, we recently described a role for Set2 in promoting dNTP synthesis in response to DNA damage and replication stress through promoting MBF-dependent transcriptional expression of *cdc22^+^*. Loss of Set2 leads to reduced Cdc22 expression, resulting in reduced dNTP levels and consequent replication stress (Pai et al., 2017). Such roles for Set2 in maintaining genome stability help explain the tumour suppressor function of the human orthologue, SETD2.

Replication stress can also arise as a result of elevated CDK activity, and cyclin E and cyclin A are frequently overexpressed in cancers (Hwang and Clurman, 2005; Yam et al., 2002). Wee1 is a negative regulator of cell cycle progression where it phosphorylates and inactivates the kinase Cdc2 (yeast) or CDK1 (mammals), thereby preventing entry into mitosis (Russell and Nurse, 1987). Inactivation of Wee1 upregulates CDK activity and promotes G2-M progression. In addition to regulating entry into mitosis, studies in mammalian cells have found that WEE1 kinase inhibition can lead to dNTP depletion through increased firing of replication origins resulting from deregulated CDK activity (Beck et al., 2012).

Synthetic lethality provides an opportunity to specifically target cancer cells (Chan and Giaccia, 2011). In this respect, previous studies using fission yeast have identified checkpoint mutants (*rad1*Δ, *rad3*Δ, *rad9*Δ, *rad17*Δ and *hus1*Δ) that are synthetic lethal with Wee1 inactivation by using a temperature-sensitive allele of Wee1, *wee1-50* (al-Khodairy and Carr, 1992; Enoch et al., 1992). These *wee1-50* checkpoint-deficient double mutants manifest a strong ‘cut’ (for ‘cell untimely torn’) phenotype in which the genetic material is mis-segregated into daughter cells, consistent with cell death arising from mitotic catastrophe (Enoch et al., 1992). Indeed, inhibitors targeting human WEE1 have been developed with the aim of promoting mitotic catastrophe in G1-S checkpoint-deficient p53 mutant cancer cells (Hirai et al., 2009). As the synthetic lethal relationship between Wee1 inactivation and loss of Chk1 is conserved in mammalian cells (Chila et al., 2015), and because inhibitors to human WEE1, ATR and CHK1 have been developed with the aim of targeting cancer cells (Dobbelstein and Sorensen, 2015; Sørensen and Syljuåsen, 2012), understanding the mechanism by which their inactivation leads to cell death is of clinical significance.

In this study, we define an evolutionarily conserved role for Wee1 in preventing replication stress through suppressing CDK-induced replication origin firing, dNTP depletion and DNA damage. Furthermore, we show that, following Wee1 inactivation, Set2-dependent histone H3K36 tri-methylation and the DNA integrity checkpoint perform an essential role in maintaining dNTP homeostasis, thus preventing replication catastrophe. These findings provide new insights into the consequences of Wee1 inactivation and its therapeutic exploitation.

## RESULTS

### Wee1 is required for efficient S-phase progression by limiting origin firing

**Fig. 1.**
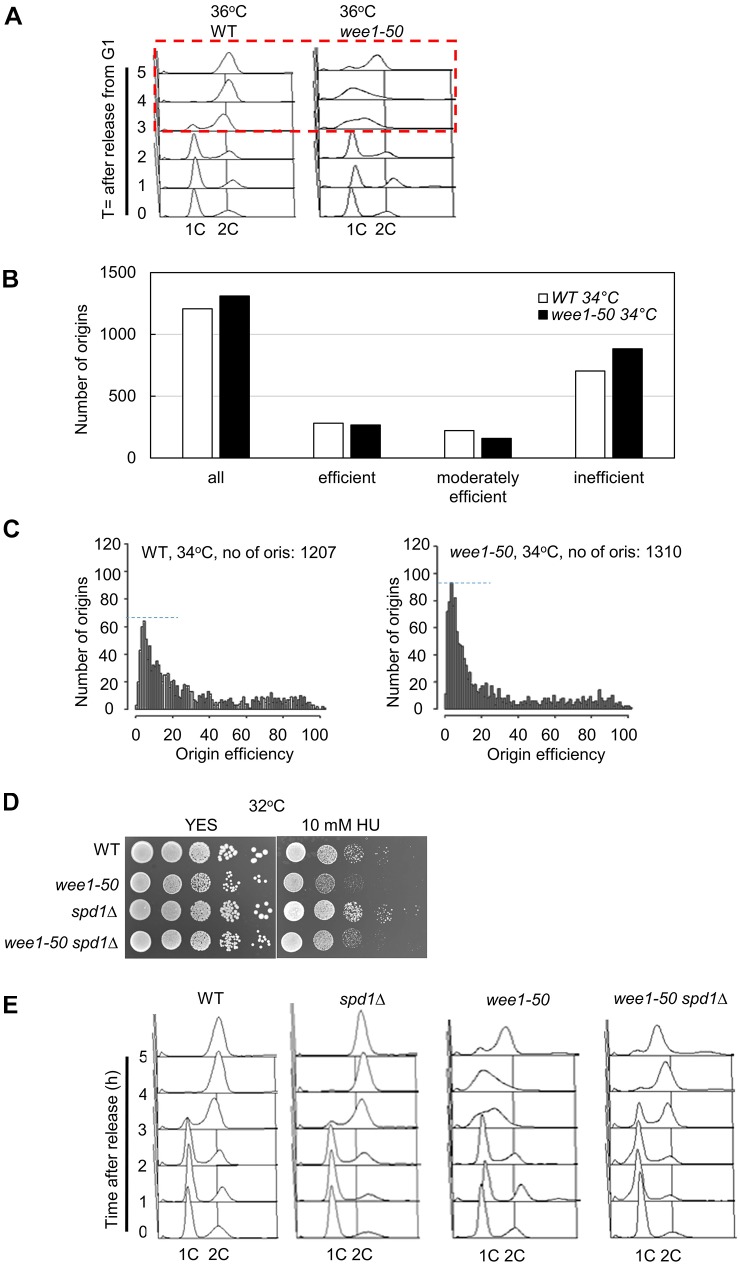
**Wee1 suppresses dormant origin firing.** (A) Wee1 is required for efficient DNA replication. Log phase WT or *wee1-50* cells were blocked in G1 phase through nitrogen starvation in EMM−N for 16 h at 25°C. Cells were released from the G1 block by re-suspending in EMM+N at 36°C. Samples were collected at the indicated time points for fluorescence-activated cell sorting (FACS) analysis. The red dashed line box indicates the delayed S-phase progression in *wee1-50* cells. (B) Wee1 suppresses firing at inefficient origins. A genome-wide plot of origin usage in *wee1-50* cells in comparison with WT cells at 34°C. Origin efficiencies were calculated from Pu-seq data. The sequencing experiment was performed once and therefore it is not possible to perform a statistical analysis. (C) The quantification of the frequency of origin usage (efficiency) in asynchronous WT and *wee1-50* cells at 34°C. The dashed blue line indicates the higher number of low-efficiency origins used in *wee1-50* cells. (D) Spd1 depletion suppresses the sensitivity of *wee1-50* cells to HU. WT and *wee1-50* cells were serially diluted and spotted onto YES plates containing 10 mM HU and incubated at 32°C for 2–3 days. (E) Deletion of *spd1^+^* promotes S-phase progression in *wee1-50* cells. WT, *spd1*Δ, *wee1-50* and *spd1*Δ *wee1-50* cells were arrested in G1 via nitrogen starvation, released and samples were taken at the time points indicated and subjected to FACS analysis.

We first investigated the possible role of Wee1 in regulating S-phase progression. Nitrogen starvation was used to synchronize *wee1-50* cells in G1 phase and, following re-feeding, cell cycle progression was monitored by flow cytometry. In wild-type (WT) cells, an increasing proportion of cells with a 2C DNA content was observed at 3 h following re-feeding; by 5 h, the entire population was 2C, indicating successful DNA replication (Fig. 1A). In contrast, in *wee1-50* cells, at 3 h after re-feeding the population exhibited a 1C peak, and even 5 h following re-feeding there was a proportion of *wee1-50* cells with a 1C peak, indicating a delay in S-phase progression (Fig. 1A, *wee1-50*).

To test whether Wee1 inactivation in fission yeast causes increased origin firing, we employed a polymerase usage sequence (Pu-seq) technique to map genome-wide origin usage as previously described (Daigaku et al., 2015). In WT cells, we identified 1207 initiation sites at 34°C including efficient (>50% usage per cell cycle), moderately efficient (25–50%) and inefficient origins (<25%) (threshold at the 20th percentile, the 99.9 percentile of all origins was set as being 100% efficient) (Fig. 1B). In the *wee1-50* background, we mapped 1310 origins at 36°C (Fig. 1B). Interestingly, analysis of the distribution of origin usage in *wee1-50* cells revealed a trend that an increased number of inefficient origins (dormant origins) were used compared to WT cells (Fig. 1B). There are a greater proportion of inefficient origins and less-efficient origins in *wee1-50* cells compared to wild type (Fig. 1C). Taken together, these data suggest that Wee1 inactivation causes an increase in the number of DNA replication initiation sites utilized.

We tested whether the increased origin firing in *wee1-50* might lead to elevated dNTP demand, thus leading to replication stress. A spot assay showed that *wee1-50* cells were sensitive to hydroxyurea (HU) at the semi-restrictive temperature (Fig. 1D; Fig. S1A). Deleting the RNR inhibitor *spd1*^+^ in a *wee1-50* background suppressed the sensitivity of *wee1-50* cells to HU (Fig. 1D; Fig. S1A) and suppressed the delayed DNA replication of *wee1-50* cells at 36°C, consistent with Wee1 inactivation having an impact on dNTP levels (Fig. 1E). Consistent with this, we showed that the dATP level (normalized to total ATP) in *wee1-50* is significantly lower than in WT cells (Fig. S1B). These findings suggest that inactivation of Wee1 causes dNTP pool depletion by increased origin firing leading to replication stress.

### Wee1 inactivation causes DNA damage accumulation and genome instability

**Fig. 2.**
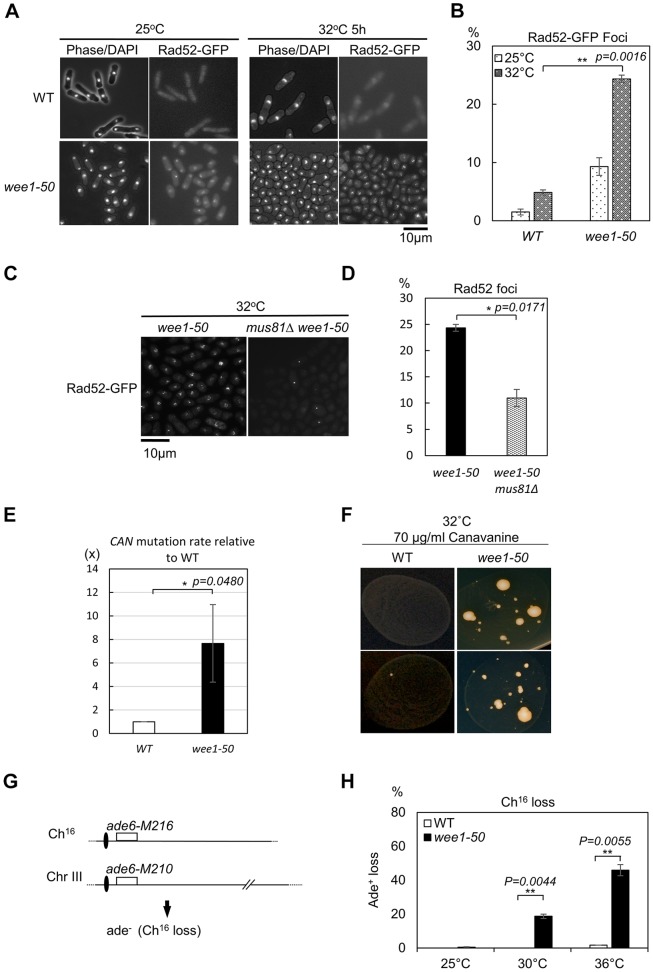
**Wee1 inactivation causes DNA damage, increases mutation rates and leads to Ch^16^ loss.** (A) Examination of Rad52–GFP foci in WT or *wee1-50* cells at 25°C or 32°C. Cells were grown to log phase at the permissive temperature before being transferred to the semi-permissive temperature for 5 h. Samples were fixed directly in methanol/acetone and examined by fluorescence microscopy. (B) The percentage of cells containing Rad52–GFP foci in the indicated strains is shown. A total of >100 cells were counted in each experimental group from two independent experiments. **P<0.01 (*t*-test). (C) A similar experiment to that described in A except a *wee1-50 mus81*Δ *rad52-GFP* strain was used. (D) Quantification analysis of *wee1-50 mus81*Δ cells with Rad52–GFP foci compared to *wee1-50* cells. A total of >100 cells were counted in each experimental group in two independent experiments *P<0.05 (*t*-test). (E) *wee1-50* cells exhibit elevated mutation rates upon canavanine treatment compared to WT cells. Cell cultures incubated on canavanine plates at 32°C for 10 days produced Can^r^ (canavanine-resistant) mutant colonies. Colony data were collected from 36 independent cultures. The mutation rates for WT and *wee1-50* strains were calculated using the MSS statistical method. The mutation rates are shown as mean for *n*≥2 experiments *P<0.05 (*t*-test). (F) Images representative of experiments performed in E at least three times. (G) Schematic of the Ch^16^ strain. Centromeric regions (ovals) and complementary heteroalleles (*ade6-M216* and *ade6-M210*) are shown for Ch^16^ and ChIII. (F) Elevated Ch^16^ loss rates associated with Wee1 inactivation. Wild-type or *wee1-50* cells containing the mini-chromosome are *ade^+^*. Cells were plated on YES or adenine-limiting plates and the percentage of Ch^16^ loss events per division was determined (*n*>500 cells for each data points). The data presented are from at least two independent biological repeats. **P<0.01 (*t*-test). All error bars are s.e.m.

We next tested whether disrupting Wee1 could lead to DNA damage associated with replication stress. We monitored DNA damage-induced Rad52 foci in a *wee1-50* mutant. Indeed, inactivation of Wee1 resulted in significantly elevated levels of DNA damage foci marked by Rad52–GFP compared to what was seen in WT cells (Fig. 2A,B). Earlier work has demonstrated that increased CDK activity promotes Mus81–Eme1 endonuclease activity (Dehé et al., 2013; Dominguez-Kelly et al., 2011). Indeed, deletion of *mus81^+^* resulted in significantly reduced levels of Rad52–GFP DNA damage foci in a *wee1-50* background (*P*<0.05) (Fig. 2C,D). Thus, Wee1 inactivation leads to elevated levels of Mus81-dependent DNA damage.

Studies in budding yeast have shown that dNTP imbalance can cause mutagenesis and induce genome instability (Kumar et al., 2011). Therefore, we tested whether Wee1 inactivation associated with DNA damage or dNTP deregulation induces mutagenesis. We used resistance to canavanine (Fraser et al., 2003; Kaur et al., 1999) to determine the mutation rate in WT and *wee1-50* backgrounds. Inactivation of Wee1 led to significantly higher mutation rates (*P*<0.05) compared to that in WT cells (Fig. 2E,F).

It is known that either increasing or decreasing origin efficiency increases the loss of minichromosome Ch^16^ owing to effects on replication fork stability (Patel et al., 2008). Consistent with this, *wee1-50* cells displayed high rates of minichromosome Ch^16^ loss at the semi-restrictive (30°C) or restrictive temperature (36°C) compared to that seen in WT cells (Fig. 2G,H). Since *spd1^+^* deletion rescued the S-phase defect in *wee1-50* cells, we expect that *spd1^+^* deletion would also suppress genome instability *in wee1-50* cells (Salguero et al., 2012). Together, these results suggest that Wee1 is essential for maintaining genome stability through suppressing replication stress, which leads to DNA damage, mutagenesis and replication fork collapse.

### Loss of Set2 methyltransferase activity is synthetic lethal with *wee1-50*

**Fig. 3.**
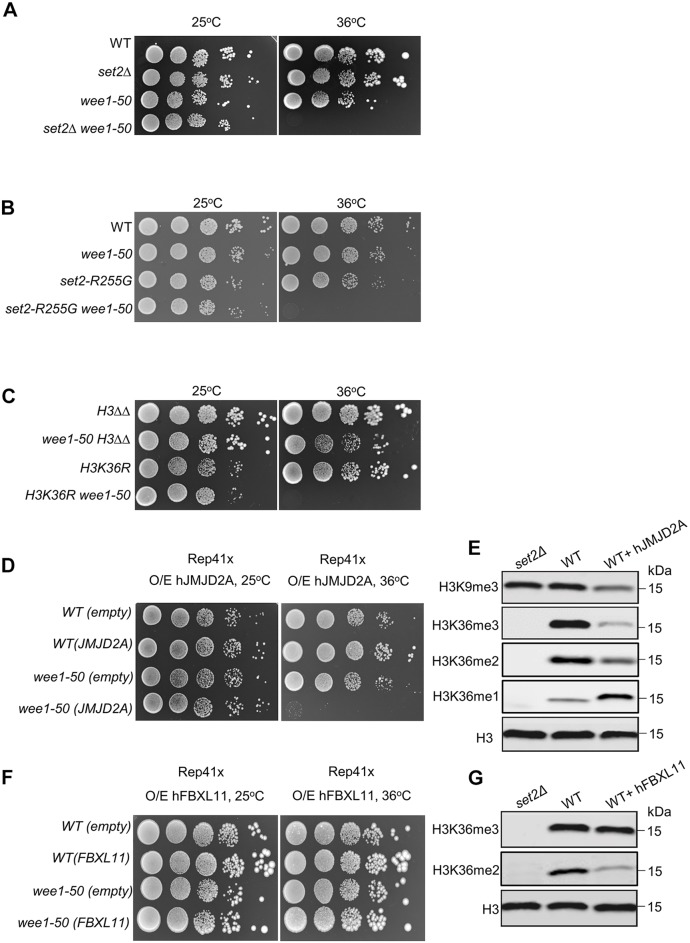
**Mutations that cause loss of Set2-dependent H3K36 methylation are synthetic lethal with *wee1-50*.** (A) WT, *set2*Δ, *wee1-50* and *set2*Δ *wee1-50* cells were serially diluted and spotted onto YES plates and incubated at the indicated temperatures for 2–3 days. (B) WT, *set2-R255G*, *wee1-50* and *set2-R255G wee1-50* cells were serially diluted and spotted onto YES plates and incubated at the indicated temperatures for 2–3 days. (C) H3ΔΔ (deletion of two of the three H3 genes), *wee1-50*, *H3K36R*, *H3K36R wee1-50* cells were serially diluted and spotted onto YES plates and incubated at the indicated temperatures for 2-3 days. (D) Serial dilutions of WT cells overexpressing (O/E) empty vector *pREP41x* or *pREP41x-JMJD2A* (encoding hJMJD2A), and *wee1-50* mutants expressing empty vector *pREP41x* or *pREP41x-JMJD2A*. Transformants were serially diluted and spotted onto EMM without leucine in the absence of thiamine at 25°C or 36°C. (E) Western blotting analysis of H3K9me3, H3K36me3, H3K36me2 and H3K36me1 in WT cells containing *pREP41x* or *pREP41x-JMJD2A*, and *set2*Δ cells. H3 is shown as a loading control. (F) Serial dilutions of WT cells overexpressing empty vector *pREP41x* or *pREP41x-FBXL11*, and *wee1-50* mutants overexpressing empty vector *pREP41x* or *pREP41x-FBXL11*. Transformants were serially diluted and spotted onto EMM without leucine in the absence of thiamine at 25°C or 36°C. (G) Western blotting analysis of H3K36me3 and H3K36me2 in WT cells expressing *pREP41x* or *pREP41x-FBXL11* and *set2*Δ cells. H3 is shown as a loading control.

Given that the histone H3K36 methyltransferase Set2 is required for DSB repair (Pai et al., 2014) and MBF-dependent transcription in response to DNA damage (Pai et al., 2017), we tested the possibility that the double mutant *set2*Δ *wee1-50* would show ‘sickness’ due to the accumulated DNA damage caused by Wee1 inactivation (Fig. 2A,B). Consistent with this, *set2*Δ and *wee1-50* were synthetic lethal when grown at the restrictive temperature (36°C) (Fig. 3A). To determine whether this synthetic lethality was dependent on the histone methyltransferase activity of Set2, *wee1-50* was crossed with a *set2* mutant (*set2-R255G*) in which the methyltransferase activity was abolished (Pai et al., 2014). The *set2-R255G wee1-50* double mutant was not viable at the restrictive temperature of 36°C (Fig. 3B), indicating that the methyltransferase activity of Set2 is required for viability in the absence of Wee1 kinase. Accordingly, *wee1-50* was also synthetic lethal with the H3 mutant *H3K36R* (Fig. 3C). Taken together, these results imply that loss of Set2-dependent H3K36 methylation is synthetic lethal with Wee1 inactivation.

### Loss of H3K36 tri-methylation is synthetic lethal with *wee1-50*

In contrast to SETD2, its human homologue, Set2 in *S. pombe* is responsible for all three forms of H3K36 methylation (mono-, di- and tri-methylation; H3K36me1, 2 and 3) and thus its loss cannot be used to distinguish between methylation states (Morris et al., 2005). We therefore investigated the consequences of expressing human (h)JMJD2A (also known as KDM4A), the human histone demethylase that catalyses the conversion of H3K36me3/me2 into H3K36me2/me1, under the control of the thiamine repressible (*nmt*) promoters (Fig. S2A,C), on a plasmid in WT or *wee1-50* cells at the permissive or restrictive temperatures (Hillringhaus et al., 2011; Klose et al., 2006; Shin and Janknecht, 2007; Whetstine et al., 2006). Overexpression of hJMJD2A was synthetic lethal with *wee1-50* at 36°C (Fig. 3D; Fig. S2E). Consistent with previous studies, expression of hJMJD2A resulted in a reduction of H3K36me3 and H3K36me2 levels (Fig. 3E). In addition to H3K36me3 loss, expressing hJMJD2A also resulted in reduced levels of H3K9me3 (Fig. 3E; Fig. S2C). However, as we did not observe synthetic lethality between deletion of *clr4^+^*, encoding the H3K9 methyltransferase, and *wee1-50* (Fig. S3A), this indicates that H3K9me3 loss is not required for cell viability in the absence of Wee1.

To distinguish between loss of H3K36me3 and H3K36me2, we expressed the WT human H3K36me2-specific demethylase human (h)FBXL11 (also known as JHDM1A and KDM2A) in WT or *wee1-50* cells (Fig. S2B,D,F) (Tsukada et al., 2006). Accordingly, we found that expression of hFBXL11 in fission yeast resulted in a significant decrease in H3K36me2 but did not affect H3K36me3 levels (Fig. 3G), indicating that hFBXL11 preferentially demethylates H3K36me2 *in vivo*. However, expression of hFBXL11 did not induce a significant viability loss in *wee1-50* cells at 36°C (Fig. 3F), and expression of hJMJD2A or hFBXL11 did not sensitize WT or *wee1-50* cells at the permissive temperature (Fig. 3D,F). Collectively, these findings provide strong evidence that the histone mark H3K36me3 is required for viability in the absence of Wee1.

### *set2*Δ synthetic lethality with *wee1-50* can be suppressed by Cdc2 inactivation

We next explored whether Wee1 inactivation leads to synthetic lethality with *set2*Δ through elevated CDK activity or through a CDK-independent function. To test this, we investigated whether we could suppress the synthetic lethality by inhibiting CDK activity. We crossed the analogue-sensitive *cdc2* mutant (*cdc2-as*) (Dischinger et al., 2008) with *set2*Δ *wee1-50* to create a *cdc2-as set2*Δ *wee1-50* triple mutant. Instead of using the ATP analogue molecule (1-NM-PP1) to inactivate Cdc2 activity, we found that the *cdc2-as* mutant exhibited modest temperature sensitivity. As shown in Fig. S4A, loss of CDK activity suppressed the growth defect of *set2*Δ *wee1-50* mutants. Furthermore, the triple mutant showed a plating efficiency of 87.5±1.5% as compared to *set2*Δ *wee1-50*, which was only 0.3±0.3% (±s.e.m.) (Fig. S4B), while *set2*Δ and *wee1-50* single mutants exhibited more than 90% plating efficiency. Collectively, these results indicate elevated CDK activity resulting from Wee1 inactivation leads to synthetic lethality in a *set2*Δ *wee1-50* background. Since *cdc2-as* showed the same phenotype as *cdc2-ts*, we would expect that the temperature-sensitive *cdc2-ts* mutant would also suppresses *set2*Δ *wee1-50* synthetic lethality.

### set2Δ synthetic lethality with *wee1-50* results from replication catastrophe

**Fig. 4.**
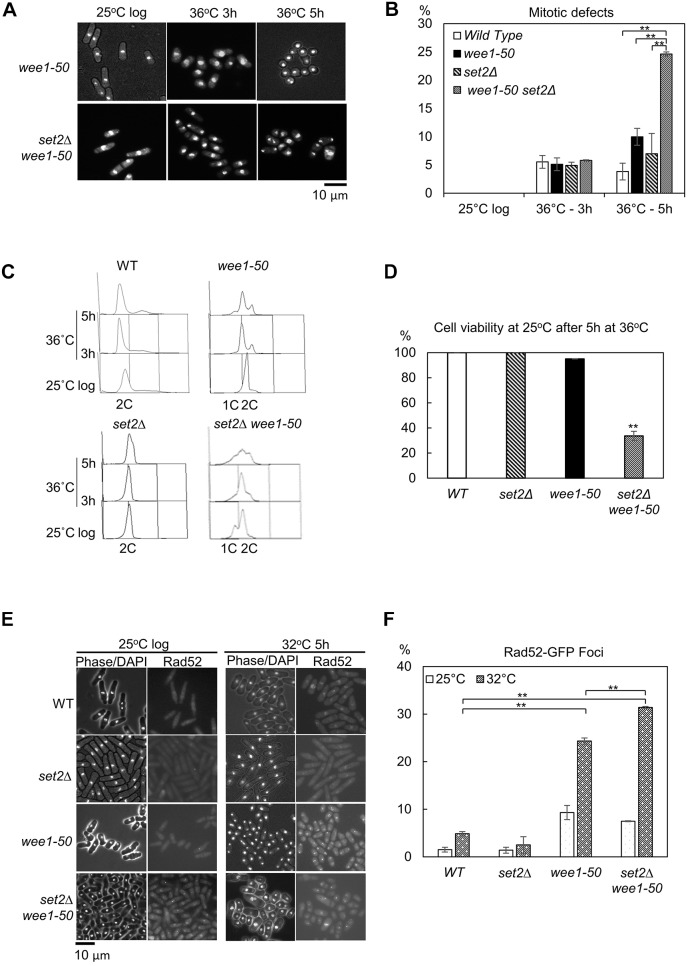
***set2***Δ **synthetic lethality with *wee1-50* results from replication catastrophe.** (A) Inactivation of Wee1 in *set2*Δ cells results in premature entry into mitosis. *wee1-50* or *set2*Δ *wee1-50* cells were grown to log phase at the permissive temperature (25°C), then incubated at 36°C to inactivate Wee1. Samples were fixed with 70% ethanol at the indicated times. The fixed cells were stained with DAPI and examined by microscopy analysis. (B) Quantitative analysis of the percentage of cells with mitotic defect cells from experiments as in A. The data presented are from at least two independent biological repeats. **P<0.01 (*t*-test, *n*≥2 experiments for each genotype, *n*>200 cells for each data point). (C) WT, *wee1-50*, *set2*Δ and *set2*Δ *wee1-50* strains were grown to log phase at the permissive temperature (25°C) then transferred to 36°C for the times shown. At the indicated times, cells were processed for FACS analysis. (D) WT, *set2*Δ, *wee1-50* and *set2*Δ *wee1-50* cells from the 5 h time point in (C) were collected, plated on the YES medium and incubated at 25°C for 3–4 days for viability analysis. (*n*≥2 experiments for each genotype, *n*>500 cells for each data point). **P<0.01 (*t*-test, between WT and *set2*Δ *wee1-50* cells; *P*-value=0.0031). (E) Examination of Rad52–GFP foci in WT, *set2*Δ, *wee1-50* and *set2*Δ *wee1-50* cells at 25°C or 32°C. Cells were grown to log phase at the permissive temperature before being transferred to the semi-permissive temperature (32°C) for 5 h. Samples were fixed directly in methanol/acetone and examined by fluorescence microscopy. (F) The percentage of cells containing Rad52–GFP foci in the indicated strains is shown. **P<0.01 (*t*-test; *n*≥2 experiments, *n*≥100 cells for each data point). All error bars are s.e.m.

Previous studies using fission yeast found that a number of checkpoint mutants (*rad1*Δ, *rad3*Δ, *rad9*Δ, *rad17*Δ and *hus1*Δ) were synthetic lethal with *wee1-50* at the restrictive temperature (al-Khodairy and Carr, 1992; Enoch et al., 1992). These double mutants exhibited a ‘cut’ phenotype suggesting that cell death arose through mitotic catastrophe (Enoch et al., 1992). Thus, we suspected that the synthetic lethality seen in *set2*Δ *wee1-50* cells might be also due to premature entry into mitosis. We found that *set2*Δ *wee1-50* cells had the ‘wee’ phenotype, and 26% of *set2*Δ *wee1-50* cells exhibited a ‘cut’ phenotype at 36°C after 5 h incubation (Fig. 4A,B). This level of cutting in *set2*Δ *wee1-50* cells was significantly higher than *wee1-50* cells (10%) (*P*<0.05) (Fig. 4A,B). Surprisingly, *set2*Δ *wee1-50* cells showed a striking S-phase delay even at the permissive temperature (Fig. 4C), suggesting inactivation of Wee1 causes more extreme DNA replication defects in *set2*Δ cells. Flow cytometry analysis showed that *set2*Δ *wee1-50* cells accumulated in S-phase following a shift to 36°C for 3–5 h (Fig. 4C). This result suggests that the observed cell death might be mostly due to a permanent replication stalling in the *set2*Δ *wee1-50* double mutant rather than through mitotic catastrophe (Fig. 4A,B). Nevertheless, 72±5% (±s.e.m.) of *set2*Δ *wee1-50* cells exhibited a ‘cut’ phenotype following a shift to 36°C for 24 h (Fig. S5A,B), suggesting that the majority of *set2*Δ *wee1-50* cells eventually undergo mitotic catastrophe after long-term replication stalling.

To investigate whether the replication arrest was the cause of synthetic lethality in *set2*Δ *wee1-50* cells, double mutants were incubated at the restrictive temperature of 36°C for 5 h and the cell viability was examined by returning them to the permissive temperature of 25°C. The results showed that 66% of *set2*Δ *wee1-50* cells lost viability after shifting to the restrictive temperature for 5 h (Fig. 4D), in which the majority of double mutants had arrested during DNA replication but only 26% of the double mutants underwent mitotic catastrophe (Fig. 4B), suggesting that most *set2*Δ *wee1-50* cells were dying in S-phase.

Furthermore, we found that *set2*Δ *wee1-50* cells exhibited elevated levels of DNA damage compared to WT cells (Fig. 4E,F), indicative of replication stress-induced DNA damage accumulation. Together, these data suggest that Wee1 inactivation in a *set2*Δ background leads to elevated levels of replication fork collapse and DNA damage.

### *set2*Δ *wee1-50* replication catastrophe results from nucleotide depletion

**Fig. 5.**
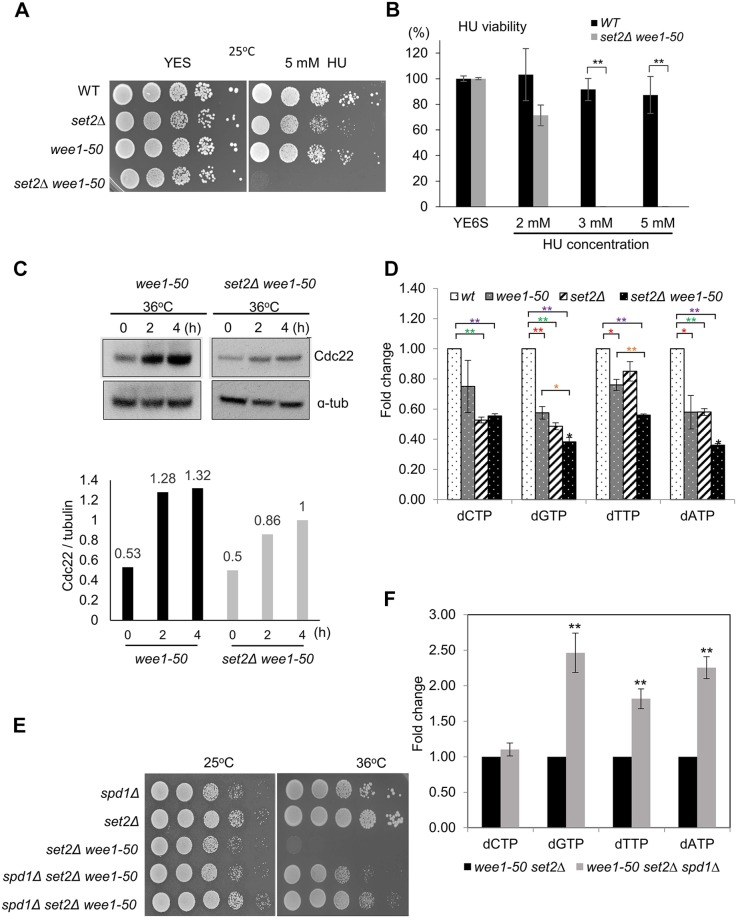
**The synthetic lethality between *set2***Δ **and *wee1-50* results from dNTP depletion.** (A) *set2*Δ *wee1-50* cells are sensitive to low levels of HU. WT, *set2*Δ, *wee1-50* and *set2*Δ *wee1-50* cells were serially diluted and spotted onto YES plates containing 5 mM HU and incubated at the permissive temperature (25°C) for 3–4 days. (B) Quantification (mean±s.e.m.) of the viability of WT and *set2*Δ *wee1-50* cells on YES plates at 25°C containing different concentrations of HU as indicated (*n*≥2 experiments, *n*≥500 cells for each data point). **P<0.01 (*t*-test). (C) The endogenous protein levels of Cdc22 were examined in *wee1-50* and *set2*Δ *wee1-50* cells following incubation at 36°C for 4 h. Samples of cells were taken at the indicated time points and cell extracts were made by using the TCA method. Cdc22 was detected using an antibody against the CFP tag. α-tubulin is shown as a loading control. The lower panel shows a quantification of Cdc22 levels in *wee1-50* and *set2*Δ *wee1-50* cells for the representative blot in the upper panel. (D) dNTP levels were measured in WT, *wee1-50*, *set2*Δ and *set2*Δ *wee1-50* strains. Cells were grown to log phase at 25°C followed by a 5 h incubation at 36°C. Samples of cells were collected and re-suspended in 10% TCA for subsequent HPLC analysis following neutralization. Means±s.e.m. of three experiments are shown. *P<0.05, **P<0.01 (*t*-test). (E) *spd1*Δ suppresses the synthetic lethality of *set2*Δ *wee1-50*. Strains were serially diluted and spotted onto YES plates and incubated at the indicated temperatures for 2–3 days. (F) dNTP levels were measured in *set2*Δ *wee1-50* and *spd1*Δ *set2*Δ *wee1-50* strains. Cells were grown to log phase at 25°C followed by a 5 h incubation at 36°C. Samples of cells were collected and re-suspended in 10% TCA for subsequent HPLC analysis following neutralization. The mean±s.e.m. for three experiments are shown. **P<0.01 (*t*-test between *set2*Δ *wee1-50* and *spd1*Δ *set2*Δ *wee1-50* strains; *P*-values: dCTP=0.3288, dGTP=0.0065, dTTP=0.0042, dATP=0.0011). The data presented are from three independent biological repeats.

Our data indicate that Wee1 inactivation leads to nucleotide depletion. Further, we have independently identified a role for Set2 in dNTP synthesis. We showed that dNTP levels were lower in *set2*Δ cells compared to wild type (Pai et al., 2017). We therefore tested the possibility that the *set2*Δ *wee1-50* synthetic lethality during S-phase was due to severe nucleotide depletion. Consistent with this, we found *set2*Δ *wee1-50* cells to be acutely sensitive to low levels of HU (Fig. 5A,B). These cells exhibited elongated phenotypes upon HU treatment, suggesting that the lethality of the double mutant was not due to the compromised checkpoint (Fig. S5C). Instead, the lethality was more likely to be due to dNTP starvation. Consistent with this, we found that Cdc22 levels (the catalytic subunit of RNR) were reduced in response to replication stress induced in a *set2*Δ *wee1-50* double mutant compared to a *wee1-50* mutant (Fig. 5C). In accordance with this observation, dNTP levels are also significantly lower in *set2*Δ *wee1-50* in comparison to *wee1-50* cells under replication stress at 36°C (*P*<0.005) (Fig. 5D). Furthermore, deleting *spd1^+^* robustly suppressed the synthetic lethality of the *set2*Δ *wee1-50* double mutant at 36°C (Fig. 5E), indicating elevated dNTPs can suppress *set2*Δ *wee1-50* synthetic lethality. Consistent with this observation, we found that dNTP levels are higher in the *spd1*Δ *set2*Δ *wee1-50* triple mutant compared to *set2*Δ *wee1-50* double mutants (Fig. 5F). Taken together, these findings indicate that the *set2*Δ *wee1-50* synthetic lethality resulted from dNTP depletion, to below a critical level.

### The nucleotide depletion in *set2*Δ *wee1-50* cells is caused by downregulation of the transcription of MBF-dependent genes and increased origin firing

**Fig. 6.**
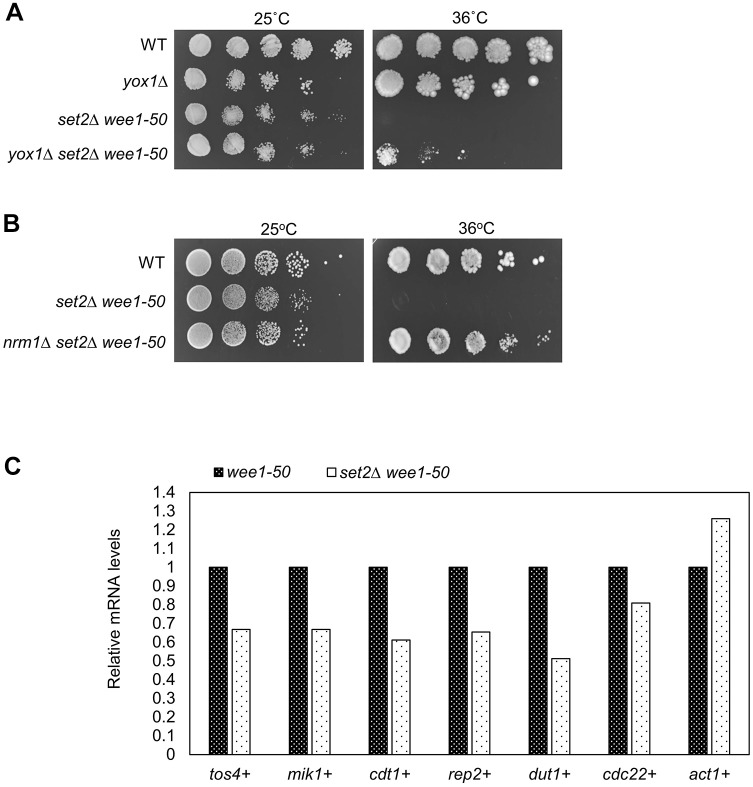
**Set2 is required for MBF-dependent gene expression in *wee1-50* cells.** (A) *yox1*Δ suppresses the synthetic lethality of *set2*Δ *wee1-50*. Strains were serially diluted and spotted onto YES plates and incubated at 25°C or 36°C for 2–3 days. (B) *nrm1*Δ suppresses the synthetic lethality of *set2*Δ *wee1-50* cells. Strains were serially diluted and spotted onto YES plates and incubated at 25°C or 36°C for 2–3 days. (C) *tos4^+^*, *mik1^+^*, *cdt1^+^*, *rep2*^+^, *dut1^+^ and cdc22^+^* transcript levels in *set2*Δ *wee1-50* cells relative to that in *wee1-50* (the expression level in *wee1-50* cells is set as 1.0). Data are the mean calculated from two biological repeats. *act1^+^* is shown as an MBF-independent control.

We have shown that Set2 controls dNTP synthesis through regulation of MBF transcription activity (Pai et al., 2017). As part of that study we found that deletion of MBF transcriptional repressor Yox1 suppressed the prolonged S-phase in *set2*Δ cells (Pai et al., 2017). Thus, we tested whether deletion of Yox1 could suppress the lethality of the *set2*Δ *wee1-50* double mutant and found that the triple mutant exhibited an increase in viability (Fig. 6A), indicating elevated MBF transcription activity can suppress *set2*Δ *wee1-50* synthetic lethality, presumably due to an increase in dNTP pools. Consistent with this, deletion of MBF transcriptional repressor Nrm1 also supressed the synthetic lethality of *set2*Δ *wee1-50* cells (Fig. 6B). Furthermore, we tested whether Set2 also affected mRNA levels of MBF-dependent genes in a *wee1-50* background. To do this, the *set2*Δ *wee1-50* double and *wee1-50* single mutants were grown at the restrictive temperature of 36°C for 5 h and global levels of gene expression were compared through microarray experiments. This analysis revealed that transcription of the MBF transcription factor activator *rep2^+^* and MBF-dependent genes *tos4^+^*, *cdt1^+^*, *mik1^+^*, *cdc22^+^* and *dut1^+^* (Aligianni et al., 2009) was reduced following *set2^+^* deletion in a *wee1-50* background, while *act1^+^*, which is not MBF-induced, was not (Fig. 6C). Taken together, these findings support a role for Set2 in facilitating MBF transcription in response to DNA damage or replication stress resulting from Wee1 inactivation, presumably through the regulation of Rep2 function in MBF activation.

Consistent with observations above (Fig. 1A), inactivation of Wee1 also leads to more origin firing in *set2*Δ cells (Fig. S6A). We also found that partial inactivation of replication licensing factor Cdc18 (*cdc18^ts^* at 34°C) suppressed the synthetic lethality of *set2*Δ *wee1-50* mutants at the semi-restrictive temperature (Fig. S6B), suggesting that reducing the number of active replication origins alleviates dNTP depletion in the *set2*Δ *wee1-50* background. No further synthetic lethality or sickness in *cdc18^ts^ wee1-50* or *mcm4tdts wee1-50* cells at the semi-restrictive temperature suggesting replication stalling is due to dNTP depletion rather than defects in other steps of DNA replication (Fig. S6C). Moreover, we did not observe synthetic sickness between Polε and Wee1 inactivation, indicating that the slow S-phase in *set2*Δ cells is unlikely to be due to the defective polymerase function (Fig. S6D).

### Disrupting checkpoint-dependent dNTP synthesis with *wee1-50* results in replication catastrophe

**Fig. 7.**
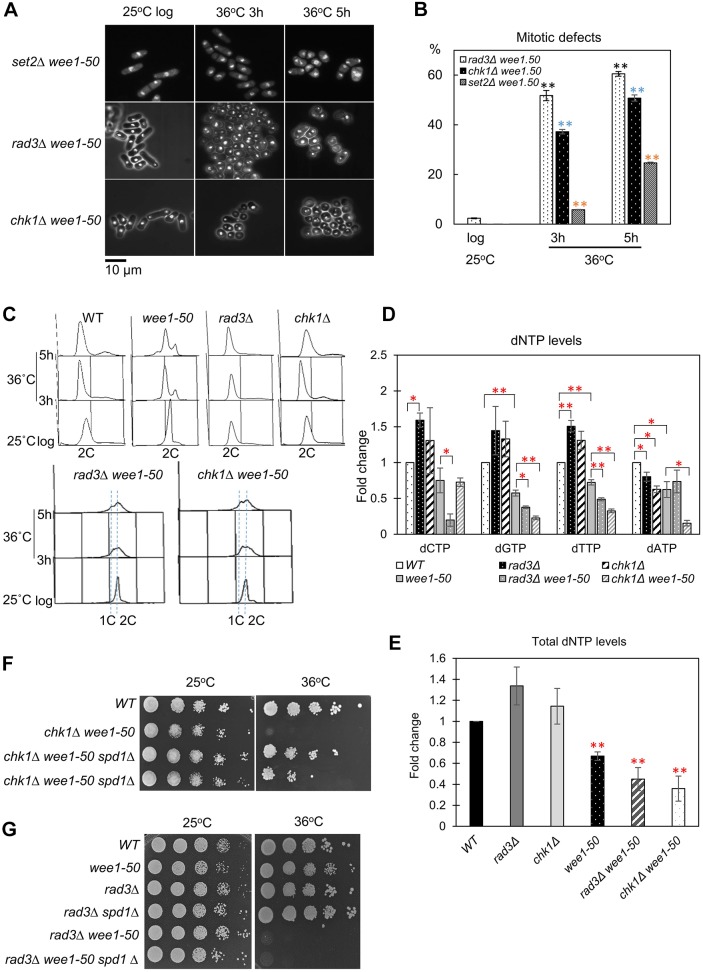
***rad3***Δ **and *chk1***Δ **are synthetic lethal with *wee1-50* through replication stress.** (A) *set2*Δ *wee1-50*, *rad3*Δ *wee1-50* or *chk1*Δ *wee1-50* result in premature entry into mitosis but the proportion of cells with the ‘cut’ phenotype in the *set2*Δ *wee1-50* mutant is significantly lower at earlier time points. *set2*Δ *wee1-50*, *rad3*Δ *wee1-50* and *chk1*Δ *wee1-50* cells were grown to log phase at the permissive temperature (25°C), then incubated at 36°C to inactivate Wee1. Samples were fixed with 70% ethanol at the indicated times. The fixed cells were stained with DAPI and examined by microscopy analysis. (B) Quantitative analysis of cells in A. Means±s.e.m. are shown. **P<0.01 (*t*-test); black asterisks indicates statistically significant differences between *rad3*Δ *wee1-50* cells grown at either 36°C or 25°C (*P*-values for *rad3*Δ *wee1-50* cells: 4h=0.0017, 5h=0.0003; averages of *n*≥2 experiments, *n*≥100 cells for each data point); blue asterisks indicate statistically significant differences between *chk1*Δ *wee1-50* cells grown at either 36°C or 25°C (*P*-values for *chk1*Δ *wee1-50* cells: 4 h=0.0006, 5h=0.0006; averages of *n*≥2 experiments, *n*≥100 cells for each data point); orange asterisks indicate statistically significant differences between *set2*Δ *wee1-50* cells grown at 36°C or 25°C (*P*-values for *set2*Δ *wee1-50* cells: 4h=0.0002, 5h=0.0078; averages of *n*≥2 experiments, *n*≥100 cells for each data point). (C) Wee1 inactivation causes replication stress in *rad3*Δ or *chk1*Δ mutants. Flow cytometric analysis of WT, *rad3*Δ, *chk1*Δ, *rad3*Δ *wee1-50* or *chk1*Δ *wee1-50* cells at 25°C or 36°C at the indicated time points. (D) dNTP levels were measured in WT, *wee1-50*, *rad3*Δ *wee1-50* and *chk1*Δ *wee1-50* strains. These strains were grown to log phase at 25°C following by a 5 h incubation at 36°C. Samples of cells were collected and re-suspended in 10% TCA for HPLC analysis. Means±s.e.m. of three biological repeats are shown. *P<0.05, **P<0.01 (*t*-test). (E) Total dNTP levels are reduced in *wee1-50*, *rad3*Δ *wee1-50* or *chk1* Δ *wee1-50* cells compared to WT cells. **P<0.01 (*t*-test; *P*-values: *wee1-50*=0.0012, *rad3*Δ *wee1-50*=0.0075, *chk1*Δ *wee1-50=*0.0059). The data presented are from three independent biological repeats. (F) *spd1*Δ suppresses the synthetic lethality of *chk1*Δ *wee1-50*. Strains were serially diluted and spotted onto YES plates and incubated at indicated temperatures for 2–3 days. (G) *spd1*Δ cannot suppress the synthetic lethality of *rad3*Δ *wee1-50*. A similar experiment was carried out as described in F.

We and others have identified a role for the DNA damage checkpoint in inducing dNTP synthesis in response to genotoxic stress (Blaikley et al., 2014; Liu et al., 2003; Moss et al., 2010). We therefore compared the effects of inactivating Wee1 in *set2*Δ cells with those seen when Wee1 was inactivated in *rad3*Δ or *chk1*Δ cells (Fig. S7A–C). We were unable to make the *cds1*Δ *wee1-50* double mutant as they were lethal at 25°C (Fig. S7D). We found that *set2*Δ *wee1-50* cells phenocopied the synthetic lethality of *rad3*Δ *wee1-50*, *hus1*Δ *wee1-50* and *chk1*Δ *wee1-50* mutants (Fig. S7). However, we found that the percentage of cells exhibiting a ‘cut’ phenotype in *set2*Δ *wee1-50* cells (20%) was significantly lower than for *rad3*Δ *wee1-50* (60.5%) *or chk1*Δ *wee1-50* (50.8%) mutants at 4 or 5 h (*P*<0.05) (Fig. 7A,B; Fig. S8). We also monitored cell cycle profiles of *rad3*Δ *wee1-50* and *chk1*Δ *wee1-50* cells following a shift to the restrictive temperature for 5 h. Surprisingly, Wee1 inactivation also caused replication stalling in *rad3*Δ and *chk1*Δ cells (Fig. 7C). In contrast, the *tel1*Δ *wee1-50* double mutant did not exhibit synthetic lethality (Fig. S3B), consistent with the fact that *tel1*Δ cells exhibit normal S-phase and DNA damage checkpoints (Willis and Rhind, 2009). Taken together, the above results indicate that disrupting Wee1 causes S-phase arrest in *set2*Δ, *rad3*Δ or *chk1*Δ cells, consistent with Wee1 playing an important role in facilitating efficient S-phase progression in fission yeast. We also examined the dNTP levels in the single and double mutants. Unexpectedly, we found that dNTP levels were increased in *rad3*Δ and *chk1*Δ cells compared to WT cells under unstressed conditions (Fig. 7D). This may reflect a lack of DNA damage checkpoint-mediated inhibition of the MBF target genes. However, deleting *rad3^+^* or *chk1^+^* in a *wee1-50* background resulted in a significant reduction in dNTP levels compared to WT or *wee1-50* cells (Fig. 7D). Total dNTP levels were significantly reduced in *wee1-50*, *rad3*Δ *wee1-50* and *chk1*Δ *wee1-50* cells compared to WT cells (Fig. 7E). Therefore, these results suggest that Rad3 and Chk1 play an important role in maintaining dNTP levels in the absence of Wee1. Furthermore, we found that it was possible to suppress the synthetic lethality following Wee1 inactivation in a *chk1*Δ background by deleting *spd1^+^* in *wee1-50 chk1*Δ cells (Fig. 7F). In contrast, deleting *spd1^+^* did not suppress the synthetic lethality of *rad3*Δ *wee1-50* double mutants (Fig. 7G), consistent with Rad3 (ATR) playing additional functions in response to replication fork stalling (Lindsay et al., 1998). These findings, together, support an essential role for Wee1 in modulating CDK-induced replication stress, and show that inactivating Wee1 together with mutations that disrupt dNTP synthesis in response to genotoxic stress results in replication catastrophe.

## DISCUSSION

Understanding the mechanisms that can lead to replication stress and how they can be targeted remains an important goal in cancer research. In this study, we define an evolutionarily conserved role for the CDK regulator Wee1 in suppressing replication stress through dNTP depletion, thereby maintaining genome stability. Furthermore, we demonstrate that mutations that cause dNTP homeostasis defects, resulting from either loss of Set2 or the DNA integrity checkpoint inactivation, are synthetic lethal with CDK-induced replication stress resulting from Wee1 inactivation. Taken together, our results support a ‘dNTP supply and demand’ model, which can be exploited to target replication stress.

Our data indicate that Wee1 inactivation leads to elevated levels of CDK-dependent replication origin firing, resulting in an overall increase in the total number of origins being fired. This in turn leads to dNTP depletion, replication stress-associated DNA damage and subsequent genome instability. We found that the replication stress associated with Wee1 inactivation alone, or in combination with *set2*Δ, resulted in DNA damage (Rad52–GFP foci formation). This DNA damage is likely to have been triggered by stalled replication forks, which present as substrates for structure-specific endonucleases (Osman et al., 2003). Consistent with this notion, elevated CDK activity has been shown to promote Mus81 activation through phosphorylation of Eme1 (Dehé et al., 2013). We hypothesize that elevated levels of CDK in the S-phase of *wee1-50* cells contribute to the Mus81-dependent DNA damage. We also observed that Wee1 inactivation resulted in robust induction of Cdc22, the catalytic subunit of RNR, thus promoting dNTP synthesis. These findings are consistent with there being a major role for Wee1 in regulating CDK activity in S-phase in fission yeast (Anda et al., 2016) and support an evolutionarily conserved role for WEE1 in regulating dNTP usage and preventing DNA damage through regulating origin firing (Beck et al., 2012). Our findings further demonstrate that Wee1 inactivation has significant consequences for genome stability.

We find Wee1 inactivation together with loss of Set2-dependent histone H3K36 tri-methylation results in synthetic lethality. Our data support a key role for Set2-dependent H3K36me3 in facilitating MBF-dependent Cdc22 transcription and thus promoting dNTP synthesis in response to genotoxic stress (Pai et al., 2017). Furthermore, Set2-dependent dNTP synthesis becomes essential following Wee1 inactivation and CDK-induced dNTP depletion. In support of this, we find loss of Set2 reduces MBF-dependent expression of Cdc22, the catalytic subunit of RNR, and leads to dNTP pool depletion in response to genotoxic stress (Pai et al., 2017). Simultaneous loss of Wee1 and Set2 leads to critically low dNTP pools and a failure to induce Cdc22 expression. This in turn leads to cell death through replicative arrest and mitotic catastrophe. Consistent with this, we find that *set2*Δ *wee1-50* synthetic lethality is associated with S-phase arrest; Cdc22 expression is significantly reduced in the double mutant compared to *wee1-50* cells; dNTP levels are significantly reduced in the double mutant compared to *wee1-50* cells, and the double mutant is acutely sensitive to HU at the permissive temperature. Accordingly, the synthetic lethality can be suppressed through increasing dNTP synthesis by depleting Spd1, by increasing MBF-dependent Cdc22 expression or by compromising replication origin licensing.

**Fig. 8.**
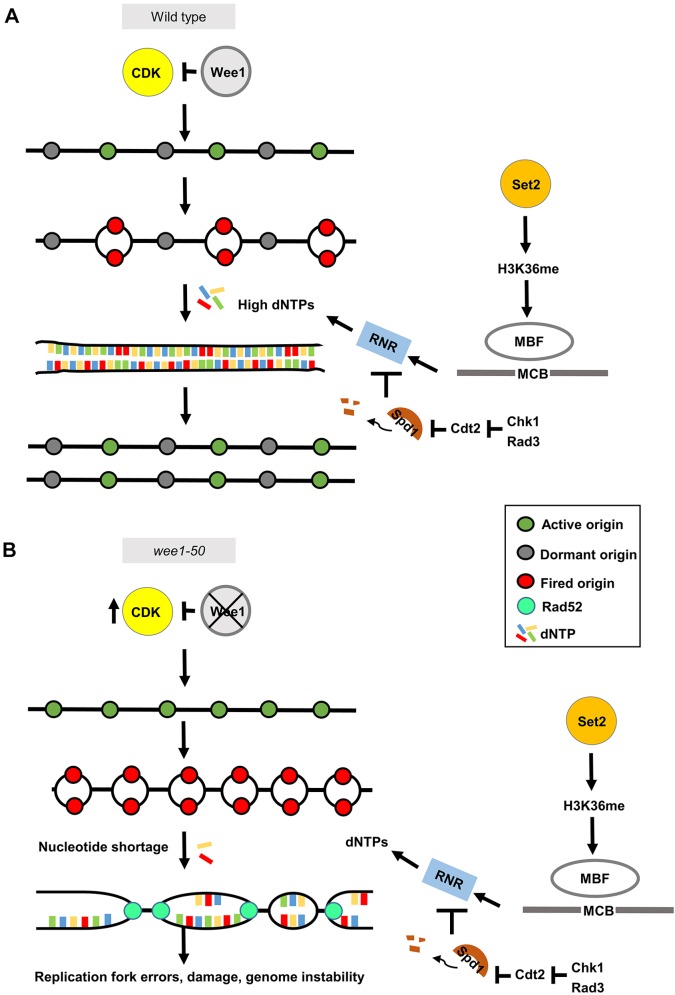
**Wee1 limits replication stress and suppresses the synthetic lethality of Set2, Rad3 (ATR) or Chk1 loss.** (A) In WT cells, Wee1, the mitotic inhibitor, controls the firing of dormant origins through limiting CDK activity during DNA replication. Set2-dependent H3K36me regulates MBF-dependent RNR expression during the cell cycle and in response to genotoxic stress. Checkpoint activation leads to degradation of Spd1 and to increased dNTP supply. Thus, Wee1, Set2 and cell cycle checkpoint proteins (Rad3 and Chk1) work together to regulate the supply of dNTP levels to limit replication stress and maintain genome stability. (B) Increased CDK activity (resulting from Wee1 inactivation) increases replication origin firing leading to increased dNTP demand. This in turn leads to replication stalling and to DNA integrity checkpoint activation. Failure to increase dNTP supply (e.g. loss of Set2, Rad3 or Chk1) when dNTP demand is high leads to replication catastrophe. See text for details.

We further define a more general role for dNTP synthesis in maintaining viability in response to Wee1 inactivation. Wee1 inactivation has been previously found to be synthetic lethal with loss of Rad3 (ATR) or Chk1 in both yeast and humans (al-Khodairy and Carr, 1992; Enoch et al., 1992; Srivas et al., 2016). Synthetic lethality between Wee1 and checkpoint-deficient mutations has been proposed to be a consequence of mitotic catastrophe. However, our results demonstrate that, while mitotic catastrophe is observed in *rad3*Δ or *chk1*Δ checkpoint mutants following Wee1 inactivation, these cells undergo prior replication arrest resulting from an insufficient dNTP supply. Importantly, the Rad3 (ATR)-dependent checkpoint pathway is required to induce dNTP synthesis following replication stress and DNA damage. DNA damage checkpoint activation leads to Cul4–Ddb1^Cdt2^-dependent degradation of Spd1, a negative regulator of RNR to promote dNTP synthesis (Moss et al., 2010). The replication checkpoint also promotes MBF-dependent transcription of Cdc22, the catalytic subunit of RNR through Cds1-dependent phosphorylation of Yox1, which blocks the binding of this negative regulator to MBF in response to replication stress (Ivanova et al., 2011). In this respect, Set2 and the DNA integrity checkpoint function analogously to facilitate dNTP synthesis in response to both DNA damage and replication stress in fission yeast. Accordingly, we show that elevating dNTP levels through deletion of *spd1^+^* suppressed the synthetic lethality of both the *set2*Δ *wee1-50* and *chk1*Δ *wee1-50* mutants. That *spd1^+^* deletion could not suppress the synthetic lethality of *rad3*Δ *wee1-50* mutant likely reflects the fact that Rad3 (ATR) performs additional roles in replication fork restart. Taken together, these findings support a ‘dNTP supply and demand’ model in which Set2 and DNA integrity checkpoint-dependent dNTP synthesis becomes essential following elevated CDK-induced origin firing and dNTP depletion, thereby preventing replication catastrophe. This model explains how Wee1 inactivation results in synthetic lethality with loss of Set2, sheds new light on the synthetic lethal relationship between loss of ATR, Chk1 and Wee1, and further predicts that other mutations that disrupt dNTP synthesis in response to replication stress will also be synthetic lethal with Wee1 inactivation (Fig. 8).

Our findings indicate that the S-M cell cycle checkpoint is intact in *set2*Δ cells (Pai et al., 2017), where an elongated phenotype is seen in response to HU or bleomycin treatment. Moreover, in contrast to *rad3*Δ or *chk1*Δ, Wee1 inactivation did not lead to rapid mitotic catastrophe in *set2*Δ cells. While *set2*Δ *wee1-50* cells underwent mitotic catastrophe at later time points, this may reflect a role for Set2 in promoting MBF-dependent transcription of *mik1^+^*, encoding the kinase Mik1, which negatively regulates Cdc2 and leads to mitotic catastrophe when deleted in a *wee1-50* background (Christensen et al., 2000; Dutta et al., 2008; Dutta and Rhind, 2009; Lee et al., 1994; Lundgren et al., 1991; Ng et al., 2001).

In accordance with the findings described here, it was demonstrated that H3K36me3-deficient human cancers are synthetic lethal with the WEE1 inhibitor AZD1775 as a result of dNTP starvation (Pfister et al., 2015). These findings are of clinical relevance as, despite the frequent loss of histone H3K36me3 in multiple cancer types and its association with poor patient outcome, there is no therapy targeting H3K36me3-deficient cancer types (Forbes et al., 2015; Lawrence et al., 2014; Li et al., 2016). Moreover, our data suggest that inhibitors of ATR and CHK1 may have different effects in cancer therapy. As inhibitors to WEE1, ATR and CHK1 are already in clinical trials (www.clinicaltrials.gov), we anticipate that our findings described here will provide important mechanistic insights into the targeting of cancers exhibiting replication stress.

## MATERIALS AND METHODS

### Yeast strains, media and genetic methods

The strains used in this study are listed in Table S1. Standard media and growth conditions were used. Cultures were grown in rich media (YE6S) or Edinburgh minimal media (EMM) at 32°C with shaking, unless otherwise stated. Nitrogen starvation was carried out using EMM lacking NH_4_Cl (EMM−N).

### Flow cytometry analysis

For flow cytometry, methanol/acetone fixed cells were rehydrated in 10 mM EDTA, pH 8.0, 0.1 mg/ml RNase A and incubated at 37°C for 2 h. Cells were then stained with 1 μM Sytox Green (ThermoFisher Scientific S7020), and analyzed using a Coulter Epics XL-MCL (Fullerton, CA).

### Serial dilution assay

A dilution series for the indicated mutant cells was spotted onto YES plates. Plates were incubated at 25°C, 32°C or 36°C for 2–3 days, as indicated, before analysis.

### Survival analysis

Exponential cultures were obtained in liquid YE6S medium inoculated with a single colony picked from a freshly streaked (YE6S) stock plate and grown overnight at 25°C with vigorous shaking. Exponential cells were resuspended in YE6S at a density of 2×10^7^ cells ml^−1^. Serial dilutions were made, and 500 cells were plated on YE6S plates at the restrictive temperature of 36°C, as well as on a control plate incubated at 25°C. Plates were incubated for 2–3 days and colonies were then scored.

### Analysis of replication origin firing

The polymerase usage sequence (Pu-seq) technique was performed as previously described (Daigaku et al., 2015). Briefly, DNA was extracted from cells grown to log phase at either 18°C or on 34°C as indicated. For ‘wt’ datasets, two strains were used, both strains containing an *rnh201* deletion together with either polymerase δ (*cdc6-L591G*) or polymerase ε (*cdc20-M630F*) mutations. These strains incorporate more rNTPs on the strands synthesized by the mutant polymerase. These sites can be mapped by Pu-seq. For the *wee1-50* and *wee1-50 set2*Δ datasets, the two strains also contained these mutations along with *rnh201* and *cdc6-L591G* or *cdc20-M630F*. The isolated DNA was then subjected to alkali treatment (0.3 M NaOH for 2 h at 55°C), which digested the DNA at the positions of rNTP incorporation and also separated the double strands. The resulting single-stranded (ss)DNA fragments were size selected on agarose gel (fragments between 300–500 bp were isolated). These fragments were then used for creating strand-specific next-generation sequencing libraries and sequenced on a Next-seq Illumina platform resulting in ∼10 M reads from each strain. The Pu-Seq data has been uploaded to Gene Expression Omnibus (GEO) under accession number GSE113747. Reads were aligned to the *S. pombe* reference sequence (http://www.pombase.org/downloads/genome-datasets), the reads were mapped using bowtie2 (http://bowtie-bio.sourceforge.net/bowtie2/index.shtml), and the data was analysed and origin positions and efficiencies were determined using the tools published and described in detail in Daigaku et al., 2015 with default variables except for the ‘percentile threshold for origins’ option was set to 0.2=20th percentile. Efficient origins were determined as origins with higher than 50% efficiency and inefficient origins had less than 25% efficiency. The sequencing experiment was performed once and therefore it is not possible to perform a statistical analysis.

### Mini-chromosome instability assay

The mini-chromosome loss assay was carried out as previously described (Allshire et al., 1995; Moss et al., 2010). Briefly, 500–1000 cells from individual *ade*^+^ colonies were plated on EMM plates containing low levels of adenine (5 mg/l), then incubated at 25°C, 30°C or 36°C for 3 days and stored for 48 h at 4°C before being scored for the presence of sectored colonies. The number of mini-chromosome loss events per division was determined as the number of Ade^−^ sectored colonies divided by the sum of white and sectored colonies. The experiment was performed in triplicate.

### The *CanR* mutation assay

To analyse mutation rates, a Luria–Delbruck fluctuation analysis was performed (Luria and Delbruck, 1943). Briefly, 1 ml cultures of WT or *wee1-50* cells were grown in YES medium to saturation in 12-well plates at 32°C. 100 µl of each culture was spotted onto PMG (−Arg, −His) plates containing 70 µg/ml canavanine and incubated at 32°C for 10–12 days. Colony numbers were scored and mutation rates in culture were analysed using the FALCOR tool (Hall et al., 2009). For each strain, colony data were collected from at least 30 independent cultures. Means and standard deviations were calculated for three independent experiments.

### Microscopy analysis

Asynchronous cell cultures were treated with 10 mM hydroxyurea (HU) at the indicated temperature before being fixed in methanol. Samples were rehydrated and stained with 4′,6-diamidino-2-phenylindole (DAPI) before examination using a Zeiss Axioplan 2ie microscope, Hamamatsu Orca ER camera and micromanager software. For visualization of Rad52–GFP foci, cells were incubated at 25°C or 32°C for 5 h before being fixed and visualized as above.

### Protein analysis

Protein extracts were made by TCA extraction and analysed by western blotting as described previously (Pai et al., 2014). TAP-tagged proteins were detected with peroxidase-conjugated anti-peroxidase soluble complex (1:1000, P1291, Sigma). Cdc22–GFP was detected using anti-GFP antibody (1:1000, 11814460001, Roche), and α-tubulin was detected with antibody T5168 (1:10,000, Sigma).

### dNTP analysis

10^8^ cells were collected and washed with 2% glucose. Cell pellets were then lysed with 50 µl 10% trichloroacetic acid (TCA) and stored at −80°C before high-pressure liquid chromatography (HPLC) analysis. On thawing, cell extracts were spun and the supernatant diluted five-fold with water. Samples were then neutralized and analysed by HPLC as described by Moss et al. using a Waters e2695 autosampler. All peak areas were measured at 258 nm (Moss et al., 2010).

### Microarray analysis

Microarray analysis was performed as previously described (Pai et al., 2014; Rallis et al., 2013). Experiments were conducted in duplicate with a dye swap. RNAs from two independent biological replicates have been utilized for cDNA production. Fig. 6C shows average expression ratios from the two repeats. Original data have been deposited in ArrayExpress under accession number E-MTAB-6795. In brief, Alexa Fluor 555- or 647-labelled cDNA was produced from the RNA, using a Superscript direct cDNA labelling system (Invitrogen) and Alexa Fluor 555 and 647 dUTP mix. cDNAs were then purified using an Invitrogen PureLink PCR Purification system and hybridized to the array using a Gene Expression Hybridization kit (Agilent). The arrays are Agilent custom-designed containing 60-mer oligonucleotides synthesized *in situ* containing 15,000 probes. Following hybridization for at least 17 h, the arrays were washed using a Gene Expression Wash Buffer kit (Agilent) and scanned in an Agilent Array Scanner. Signals were extracted using GenePix software.

## Supplementary Material

Supplementary information
