# Supplementary material for: An essential role for dNTP homeostasis following CDK-induced replication stress
Source: J Cell Sci. 2019 Mar 25;132(6):jcs226969. doi: 10.1242/jcs.226969 (PMC6451416; doi:10.1242/jcs.226969)
Supplement: Supplementary information [file joces-132-226969-s1.pdf]

a.

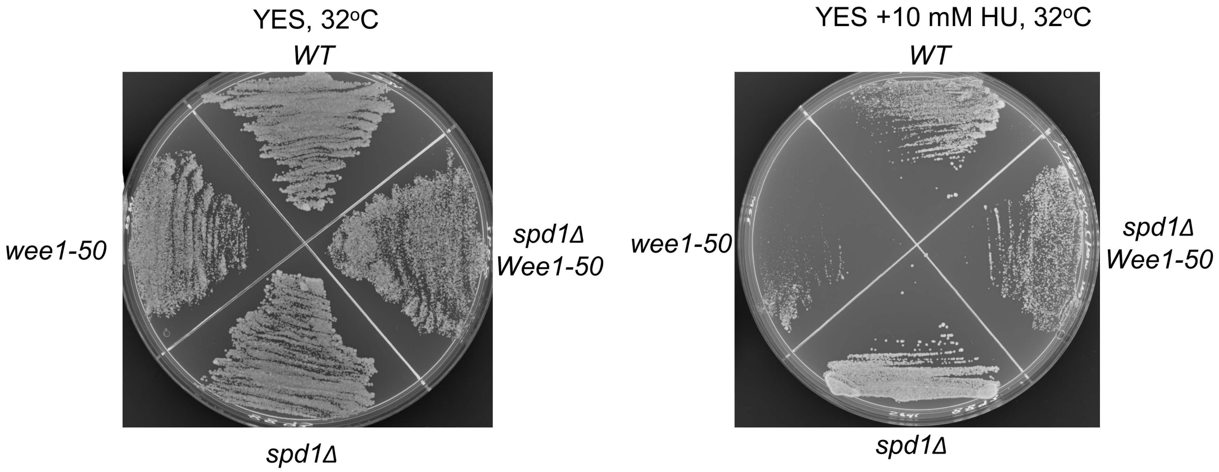

b.

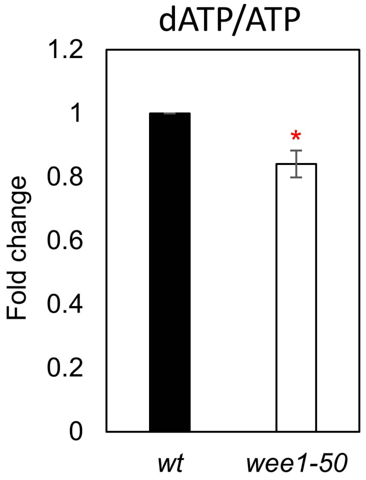

Figure S1 *wee1-50* cells are sensitive to HU. (a) Equal amount of cells were streaked on YES or 10 mM HU and incubated at 32°C. (b) dATP levels in *wee1-50* cells were significantly lower than wild-type cells. dATP levels were normalised with ATP levels. The asterisk (*) represents significant difference compared with wild type and *wee1-50* ($p < 0.05$, t test).

Journal of Cell Science • Supplementary information

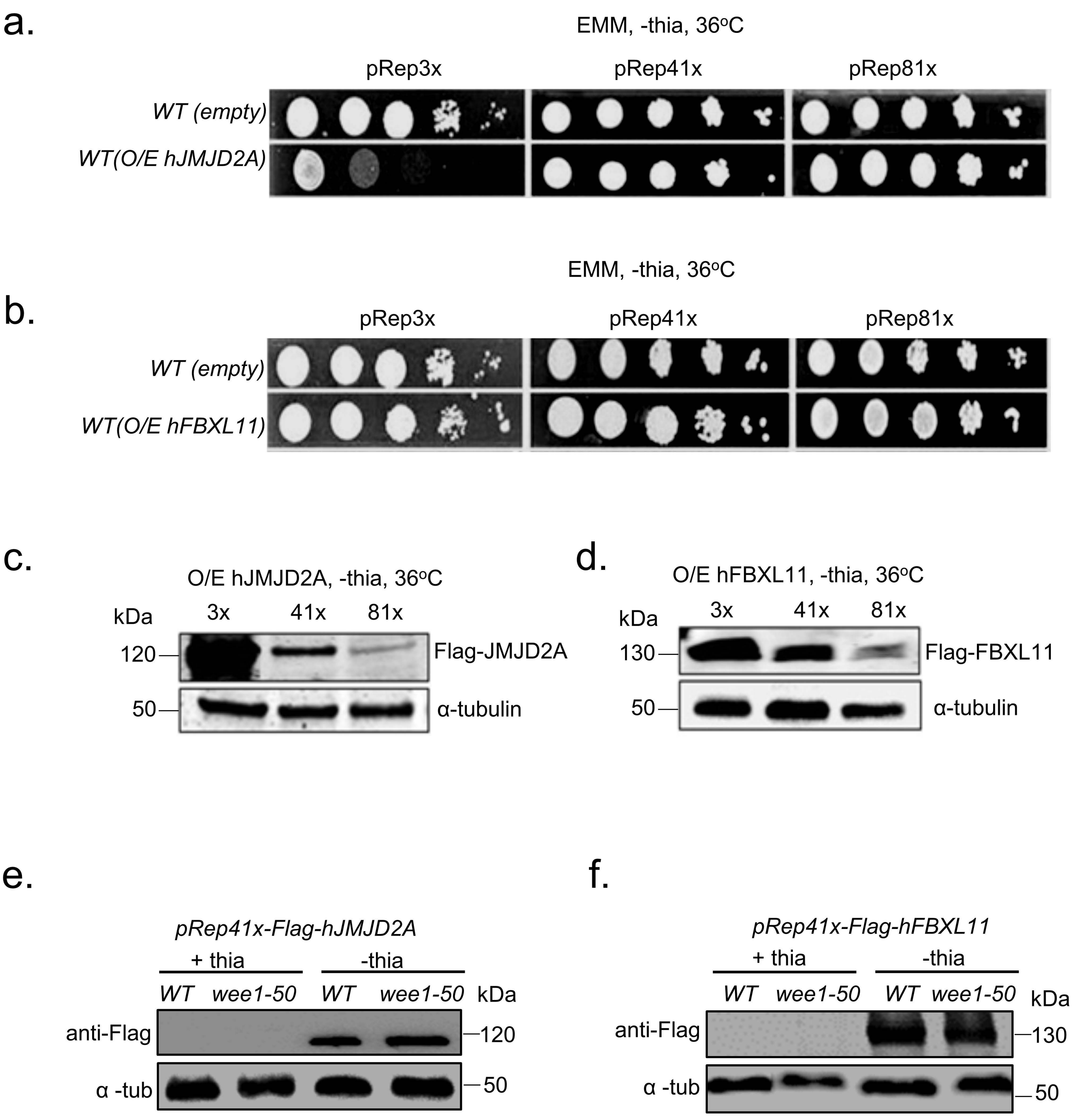

Figure S2 Expression of different levels of human histone demethylase in *S. pombe*. (a) 10-fold serial dilutions of wild type expressing empty vector, hJMJD2A using three different *nmt* promoters were spotted onto EMM minus leucine without thiamine at 36°C. (b) 10-fold serial dilutions of wild type expressing empty vector, hFBXL11 using three different *nmt* promoters were spotted onto EMM minus leucine without thiamine at 36°C. (c) Western blot analysis of overexpression of FLAG-hJMJD2A levels in wild-type cells under the control of *pREP3X*, *pREP41X* and *pREP81X* plasmids. Anti-tubulin as a loading control. (d) Western blot analysis of overexpression of FLAG-hFBXL11 levels in wild-type cells under the control of *pREP3X*, *pREP41X* or *pREP81X* plasmid. Anti-tubulin is shown as a loading control. (e) Western blot analysis of hJMJD2A levels in wild-type or *wee1-50* cells containing *pREP41x-Flag-FBXL11* plasmids. α-tubulin is shown as a loading control. (f) Western blotting analysis of hFBXL11 levels in wild-type or *wee1-50* cells containing *pREP41x-Flag-FBXL11* plasmids. α-tubulin is shown as a loading control.

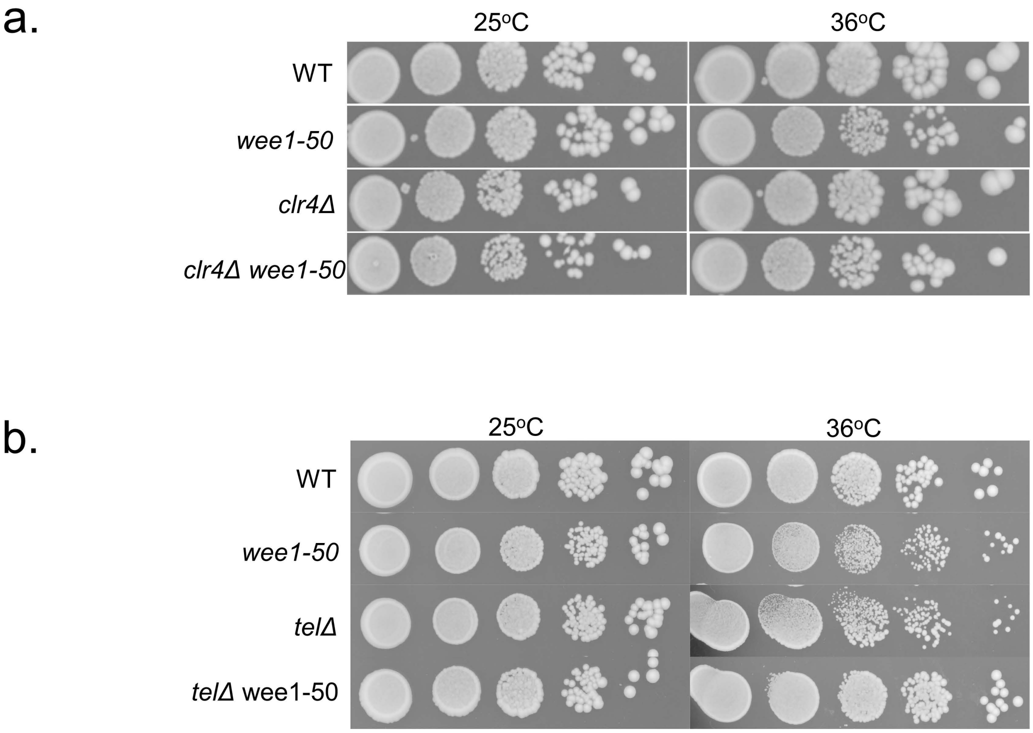

Figure S3 Loss of Clr4 or Tel1 is not essential for the viability of *wee1-50* cells. (a) Serial dilution of a wild-type, *wee1-50, clr4Δ* and *clr4Δ wee1-50* strains were spotted onto YES medium and incubated at indicated temperatures for 2-3 days. (b) *tel1Δ* is not synthetic lethal with *wee1-50*. Serial dilution of a wild-type, *tel1Δ*, *wee1-50* and *tel1Δ wee1-50* strains were spotted onto YES medium and incubated at 25°C or 36°C for 2-3 days.

a.

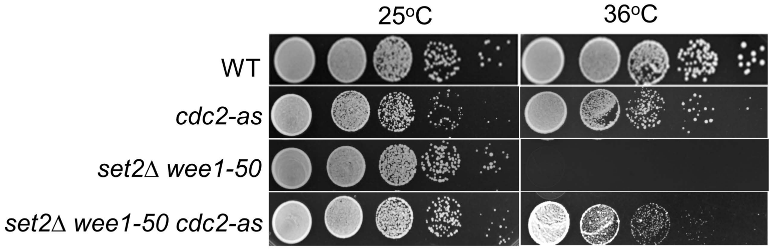

b.

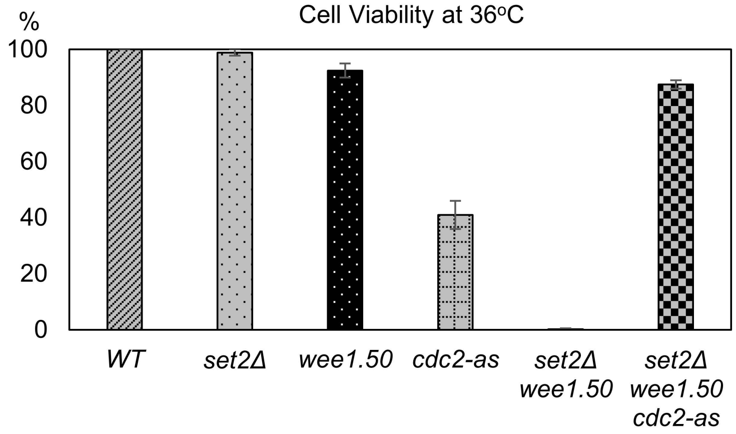

Figure S4 Loss of Cdc2 function rescues the synthetic lethality of *set2Δ wee1-50* cells. (a) Serial dilution of a wild-type, *cdc2-as, set2Δ wee1-50* and *set2Δ wee1-50 cdc2-as* strains were spotted onto YES medium and incubated at indicated temperatures for 2-3 days. (b) Quantification of the cell viability of indicated strains at 36ºC.

Journal of Cell Science • Supplementary information

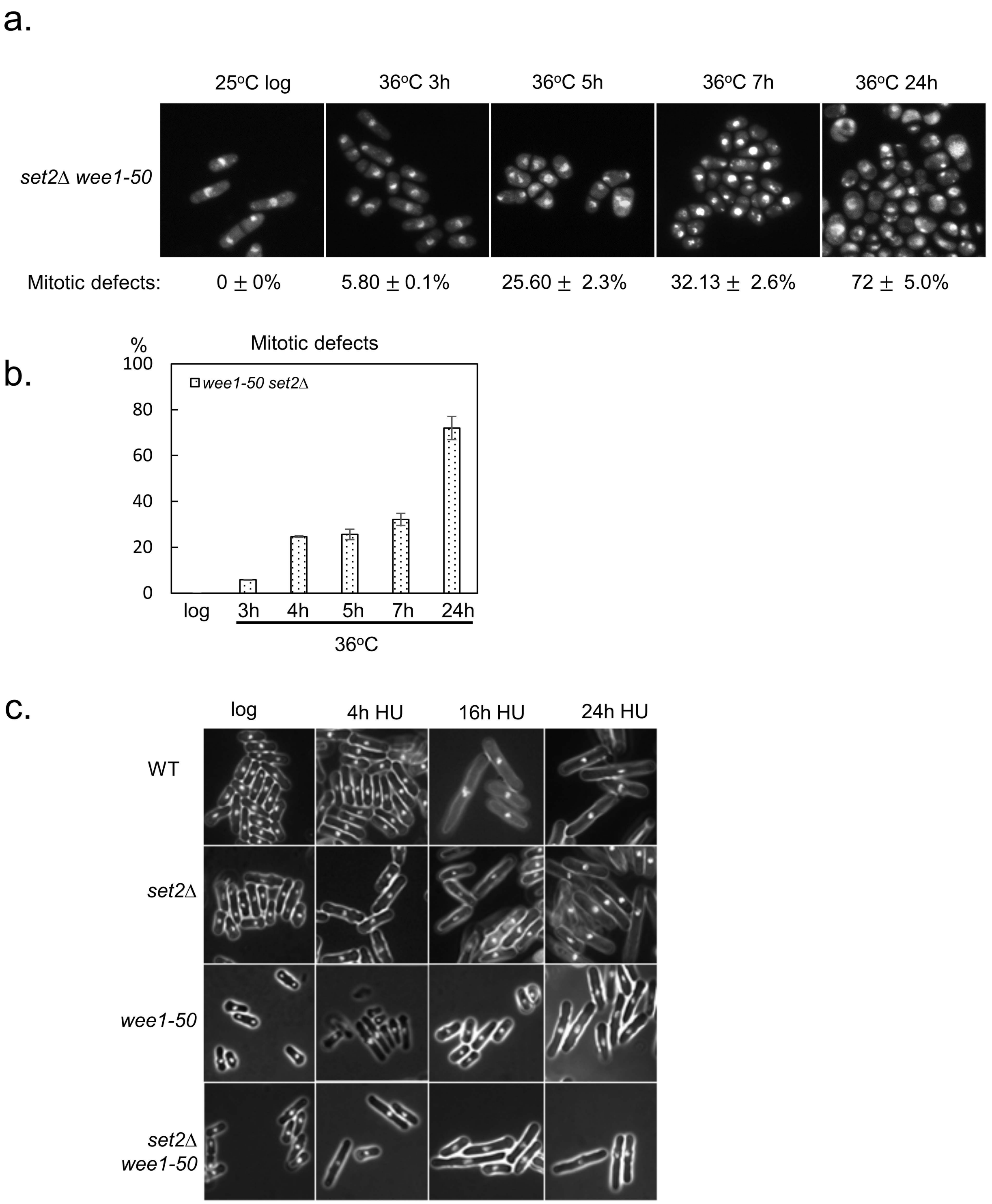

Figure S5 *set2Δ wee1-50* cells exhibit severe mitotic catastrophe at later time points following Wee1 inactivation. (a) *set2Δ wee1-50* cells were grown to log phase at the permissive temperature then transferred to the restrictive temperature for 24h. Samples of cells were collected at indicated time points and stained with DAPI for microscopy analysis. (b) Quantification of mitotic defects in (a). (c) *set2Δ wee1-50* cells exhibit proficient checkpoint activation in response to HU. Cells were grown asynchronously in YES medium and transferred to YES in the present of 10mM HU for 24h at 25°C. Samples were taken at the indicated time points and fixed with methanol/acetone; subsequently the fixed cells were examined by microscopy analysis.

Journal of Cell Science • Supplementary information

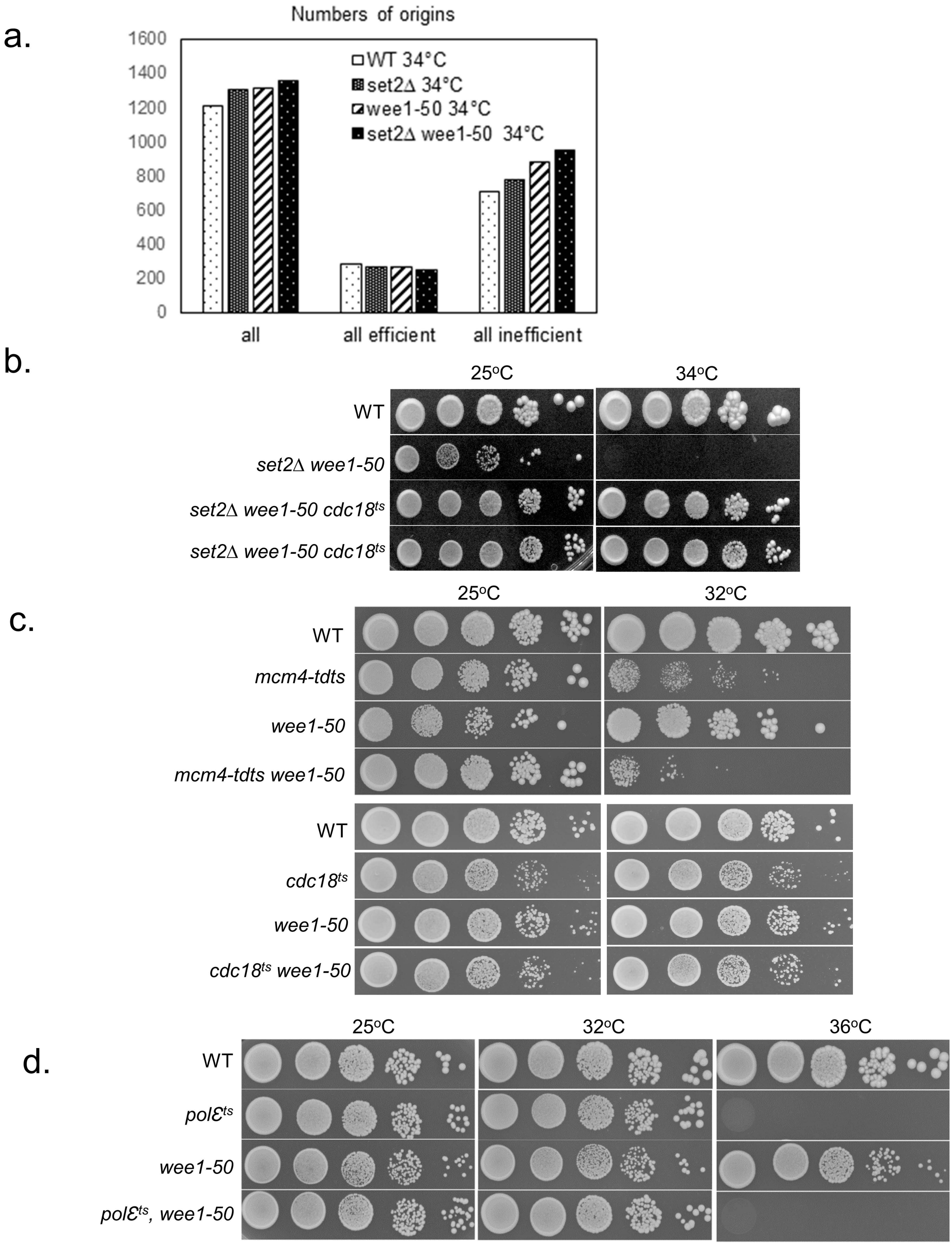

Figure S6 Inhibition of origin firing suppresses the synthetic lethality of *set2Δ wee1-50* cells. (a) Set2 and Wee1 suppresses inefficient origin firing. The genome-wide plot of origin usage in vegetative wild-type, *set2Δ, wee1-50* or *set2Δ wee1-50* cells at 34°C. Origin efficiencies were calculated from Pu-seq data. (b) Inactivation of Cdc18 at the semi-restrictive temperature suppresses the synthetic lethality of *set2Δ wee1-50* cells (c) Inhibition of replication factors Cdc18 or Mcm4 does not cause severe viability loss with Wee1 inhibition in *S. pombe*. $cdc18^{ts}$ or *mcm4-tdts* is not synthetic lethal with *wee1-50*. (d) $pol\mathcal{E}^{ts}$ is not synthetic lethal with *wee1-50*.

Journal of Cell Science • Supplementary information

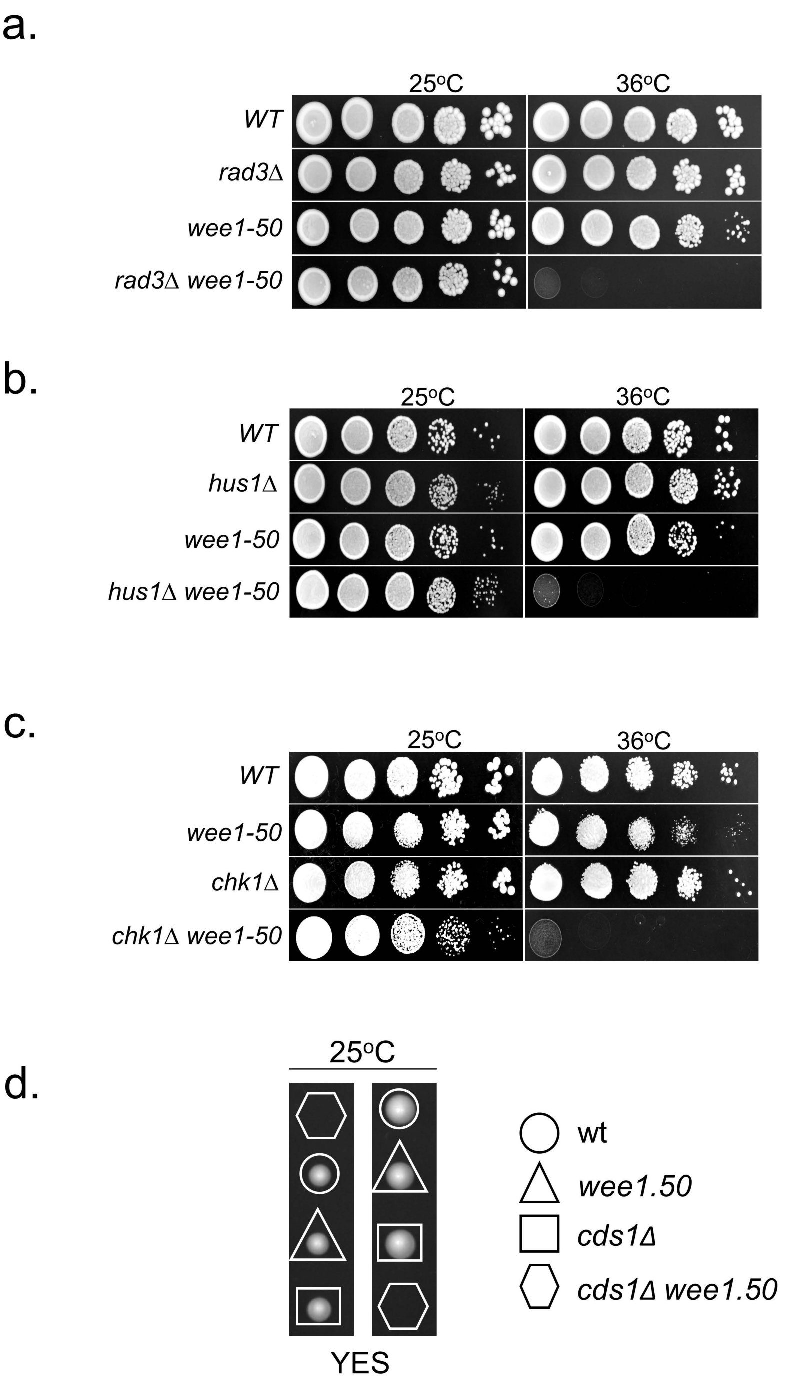

Figure S7 Checkpoint mutant is synthetic lethal with *wee1-50* mutant. (a) *rad3Δ* is synthetic lethal with *wee1-50*. Serial dilution of a wild-type, *rad3Δ*, *wee1-50* and *rad3Δ wee1-50* strains were spotted onto YES medium and incubated at indicated temperatures for 2-3 days. (b) *hus1Δ* is synthetic lethal with *wee1-50*. Similar experiments were carried as described in (a). (c) *chk1Δ* is synthetic lethal with *wee1-50*. Similar experiments were carried as described in (a). (d) *cds1Δ* is synthetic lethal with *wee1-50* at 25°C.

Journal of Cell Science • Supplementary information

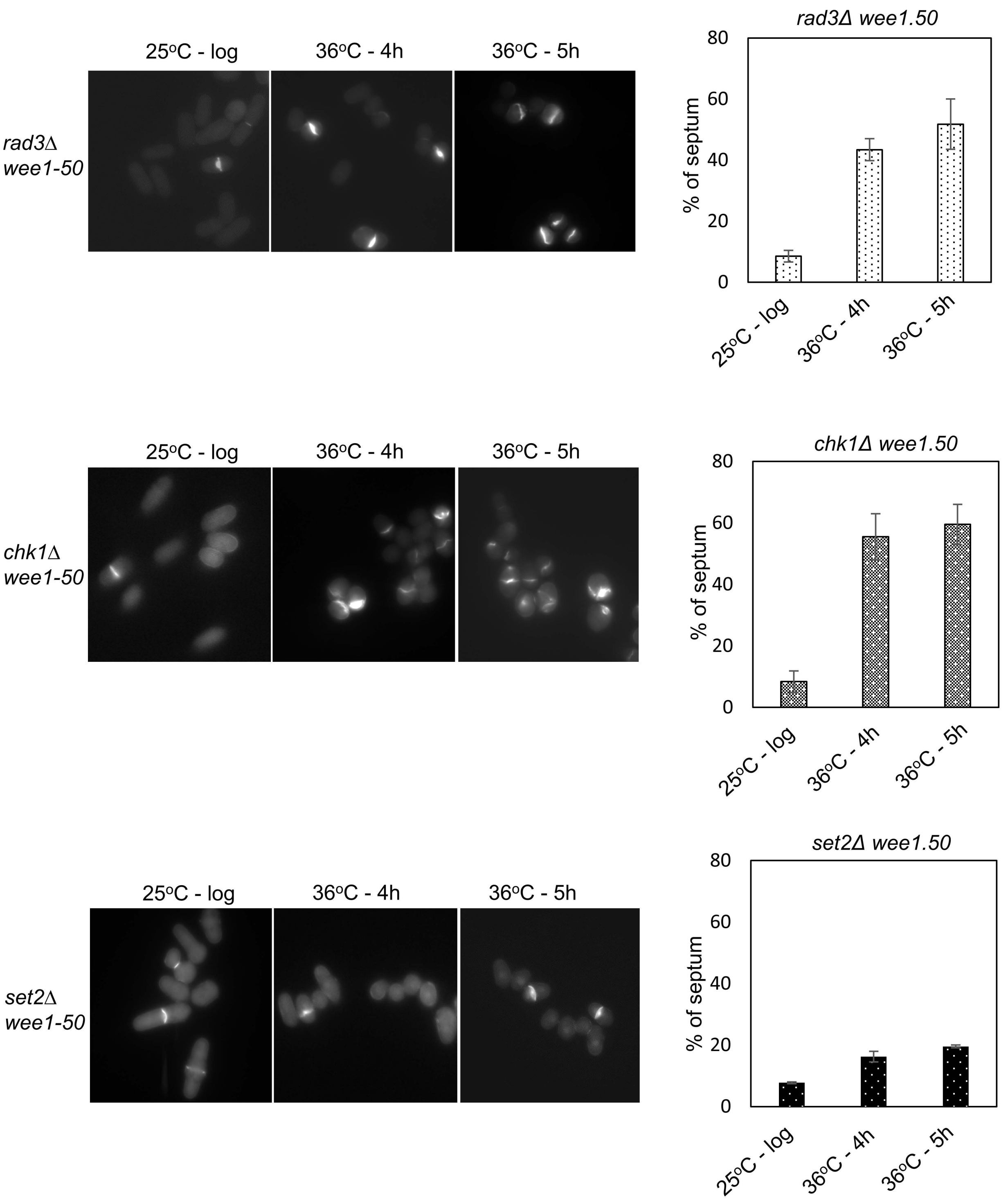

Figure S8 Wee1 inhibition causes pre-mature entry into mitosis in checkpoint mutants. Percentage of septated cells flowing Wee1 inhibition in *rad3Δ*, *chk1Δ* or *set2Δ* cells.

Table S1. Strains used in this study

Strain	Genotype	Source
TH 344	*wee1-50 h-*	Lab stock
TH 345	*wee1-50 h+*	Lab stock
TH 350	*ade6-M216 ura4-D18 leu1-32 h+*	Lab stock
TH 358	*wee1-50 leu1-32*	
TH 805	*Chr16 ade6 - M216 rad 21::mata::kanMx Chr3 ade6-M210*	Lab stock
TH 1142	*mcm4(cdc21)-tdts::ura4+leu1-32 ura4-D18 h-*	Stephen Kearsey
TH 1501	*tel1::ura4 ade6- his3D leu1-32, ura4-D18 h+*	Lab stock
TH 2094	*arg3-D4, ade6-D1, ura4-D18, leu1-32, his3-D1 h-*	Lab stock
TH 2148	*rad22::rad22-GFP::kanMx*	Miguel G Ferreira
TH 3645	*spd1::hygroR ura4-D18 leu1 ade6-704 h-*	Olaf Nielsen
TH 3271	*set2::ura4+ ade6-210 arg3-D4 his3-D1 leu1-32 ura4-D18 h-*	Robin Allshire
TH 4027	*leu1-32 ura4-D18 cdc2-as::HygR*	Lab stock
TH 5512	*yox1::kanMx arg3-D4, ade6-D1, ura4-D18, leu1-32, his3-D1 cycR*	Lab stock
TH 6236	*set2::kanMX ade6-210 leu1-32 ura4-D18 h-*	Robin Allshire
TH 6237	*set2::kanMX ade6-210 leu1-32 ura4-D18 h+*	Robin Allshire
TH 6238	*H3.1/H4.1::his3 H3.3/H4.3::arg3 ura4-DS/E ade6-M210 leu1-32 his3-D1 arg3-D4 h-*	Robin Allshire
TH 6241	*H3.2K36R H3.1/H4.1::his3 H3.3/H4.3::arg3 ura4-DS/E ade6-M210 leu1-32 his3-D1 arg3-D4 h-*	Robin Allshire
TH 6707	*set2::ura4 cdc25-22*	This study
TH 6877	*972 h-*	Lab stock
TH 6960	*cdc18-K46*	Paul Nurse
TH 7121	*set2::kanMX wee1-50 ade6-D1 ura4-D18 leu1-32 his3-D1*	This study
TH 7133	*set2::set2-R255G-ura4*	Lab stock
TH 7936	*yox1::kanMx wee1-50*	This study
TH 7961	*set2::set2-R255G-ura4 wee1-50*	This study
TH 7972	*wee1-50 spd1::hygroR arg3-D4, ura4-D18, leu1-32, his3-D1*	This study
TH 8001	*set2::kanMx mus81::kanMx wee1-50 ade6-210 ura4-D18*	This study

Journal of Cell Science • Supplementary information

TH 8064	*set2::kanMx mus81::kanMx wee1ts*	This study
TH 8073	*set2::ura4 mus81::kanMx*	This study
TH 8261	*mcm4-tdts-ura4+ wee1-50*	This study
TH 8306	*cdc2-as-hygR wee1-50 set2::kanMx*	This study
TH 8434	*set2::kanMx yox1::kanMx wee1-50*	This study
TH 8459	*set2::kanMX spd1::HygroR*	This study
TH 8491	*spd1::hygb set2::kanMx wee1-50*	This study
TH 8492	*spd1::hygb set2::kanMx wee1-50*	This study
TH 8520	*cdc18-K46 wee1-50*	This study
TH 8522	*pole^{ts} wee1-50*	This study
TH 8593	*cdc22-CFP-kanMx set2::kanMx*	This study
TH 8653	*set2::kanMx wee1-50 rad22-GFP-HYG*	This study
TH 8663	*wee1-50 cdc22-CFP-kanMx*	This study
TH 8673	*wee1-50 set2::kanMx cdc22-cfp-kanMx*	This study
TH 8701	*wee1-50 rad22-GFP::HygR*	This study
TH 8702	*set2::kanR rad22-GFP::HygR*	This study
TH 8726	*set2::kanMX cdc22-D57N*	This study
TH 8727	*cdc22-CFP:: kanMX*	Stephen Kearsey
TH 8729	*set2:: kanMX cdc22-CFP:: kanMX*	This study
TH 8930	*arg3-D4, ade6-D1, ura4-D18, leu1-32, his3-D1 pREP3X*	This study
TH 8931	*ade6-M216 ura4-D18 leu1-32 pREP41X*	This study
TH 8932	*ade6-M216 ura4-D18 leu1-32 pREP81X*	This study
TH 8933	*arg3-D4, ade6-D1, ura4-D18, leu1-32, his3-D1 pREP3X-JMJD2A*	This study
TH 8934	*arg3-D4, ade6-D1, ura4-D18, leu1-32, his3-D1 pREP41X-JMJD2A*	This study
TH 8935	*arg3-D4, ade6-D1, ura4-D18, leu1-32, his3-D1 pREP81X-JMJD2A*	This study
TH 8940	*arg3-D4, ade6-D1, ura4-D18, leu1-32, his3-D1 pREP41X-FBXL11*	This study
TH 8941	*arg3-D4, ade6-D1, ura4-D18, leu1-32, his3-D1 pREP81X-FBXL11*	This study
TH 8956	*wee1-50 leu1-32 pREP41X*	This study

Journal of Cell Science • Supplementary information

TH 8957	*wee1-50 leu1-32 pREP81X*	This study
TH 8959	*wee1-50 leu1-32 pREP41X-JMJD2A*	This study
TH 8960	*wee1-50 leu1-32 pREP41X-JMJD2A*	This study
TH 8965	*wee1-50 leu1-32 pREP41X- FBXL11*	This study
TH 8966	*wee1-50 leu1-32 pREP41X- FBXL11*	This study
TH 8990	*wee1-50 rad3::kanMx*	This study
TH 8991	*wee1-50 chk1::ura4*	This study
TH 8993	*mus81::kanMx wee1-50 rad22-GFP-hygb*	This study
TH 8994	*spd1::hygb chk1::ura4 wee1-50*	This study
TH 8995	*spd1::hygb chk1::ura4 wee1-50*	This study
TH 8996	*nrm1::HYG set2::kanMx wee1-50*	This study
TH 8997	*nrm1::HYG set2::kanMx wee1-50*	This study
TH 8998	*wee1-50 ade6-M210 Chr16 ade6 - M216 rad 21::mata-kanMx*	This study
TH 8999	*clr4::kanMx wee1-50*	This study
TH 9000	*hus1::leu+ wee1-50*	This study
TH 9001	*tel1::ura4 wee1-50 ura4-D18*	This study
TH 9002	*rad3::kanMx wee1-50 spd1::HYG*	This study
TH 9003	*cdc18-K46 set2::kanMx wee1-50*	This study
TH 9004	*cdc18-K46 set2::kanMx wee1-50*	This study
IM 655	*rnh201::KanMX cdc20-M603F::lox ade6-704 leu1-32 ura4-D18 h-*	Tony Carr
IM 856	*rnh201::KanMX cdc6-L591G:lox ade6-704 leu1-32 ura4-D18 h-*	Tony Carr
YAK250	*set2::kanMx rnh201::HYG, cdc20-M603F ade6-704 leu1-32 ura4-D18 h-*	Tony Carr
YAK251	*set2::kanMx rnh201::HYG, cdc6-L591G ade6-704 leu1-32 ura4-D18*	Tony Carr
YAK313	*set2::kanMx wee1-50 rnh201::HYG, cdc20-M603F ade6-704 leu1-32 ura4-D18 h-*	Tony Carr
YAK315	*set2::kanMx wee1-50 rnh201::HYG, cdc20-M603F ade6-704 leu1-32 ura4-D18 h-*	Tony Carr

Journal of Cell Science • Supplementary information
